# Open-source RNA extraction and RT-qPCR methods for SARS-CoV-2 detection

**DOI:** 10.1371/journal.pone.0246647

**Published:** 2021-02-03

**Authors:** Thomas G. W. Graham, Claire Dugast-Darzacq, Gina M. Dailey, Xammy Huu Wrynla, Erik Van Dis, Meagan N. Esbin, Abrar Abidi, Sarah A. Stanley, Xavier Darzacq, Robert Tjian

**Affiliations:** 1 Department of Molecular and Cell Biology, University of California Berkeley, Berkeley, California, United States of America; 2 Division of Infectious Diseases and Vaccinology, School of Public Health, University of California Berkeley, Berkeley, California, United States of America; 3 The Howard Hughes Medical Institute, University of California Berkeley, Berkeley, California, United States of America; Cairo University, EGYPT

## Abstract

Re-opening of communities in the midst of the ongoing COVID-19 pandemic has ignited new waves of infections in many places around the world. Mitigating the risk of reopening will require widespread SARS-CoV-2 testing, which would be greatly facilitated by simple, rapid, and inexpensive testing methods. This study evaluates several protocols for RNA extraction and RT-qPCR that are simpler and less expensive than prevailing methods. First, isopropanol precipitation is shown to provide an effective means of RNA extraction from nasopharyngeal (NP) swab samples. Second, direct addition of NP swab samples to RT-qPCRs is evaluated without an RNA extraction step. A simple, inexpensive swab collection solution suitable for direct addition is validated using contrived swab samples. Third, an open-source master mix for RT-qPCR is described that permits detection of viral RNA in NP swab samples with a limit of detection of approximately 50 RNA copies per reaction. Quantification cycle (Cq) values for purified RNA from 30 known positive clinical samples showed a strong correlation (r^2^ = 0.98) between this homemade master mix and commercial TaqPath master mix. Lastly, end-point fluorescence imaging is found to provide an accurate diagnostic readout without requiring a qPCR thermocycler. Adoption of these simple, open-source methods has the potential to reduce the time and expense of COVID-19 testing.

## Introduction

The current global pandemic of SARS-CoV-2 has now infected an estimated 98 million people worldwide and claimed over 2.1 million lives (Worldometer, https://www.worldometers.info/coronavirus/, accessed 1-22-2021). However, the true number of cases is likely to be even higher, and a full understanding of the scope of the pandemic has been hindered by a persistent lack of widespread testing. The U.S. Centers for Disease Control and Prevention “gold standard” test for COVID-19 detects SARS-CoV-2 viral RNA purified from patient nasopharyngeal swabs. Researchers and clinicians aiming to implement RT-PCR testing for COVID-19 have faced a shortage of the necessary reagents to perform tests in addition to the long processing times required for each test [1]. It has been argued that assays that are less sensitive yet more widely available may be more useful than exquisitely sensitive tests with limited availability [2]. The use of inexpensive, readily procurable reagents and the optimization of rate-limiting steps such as RNA extraction would help to increase the availability of tests and reduce their turnaround time.

Many current RT-PCR protocols for COVID-19 detection, including the CDC-approved test, employ an RNA extraction step to isolate and concentrate viral RNA from patient nasopharyngeal swabs prior to amplification. Typically, this involves the use of a column-based extraction kit such as the Qiagen QIAmp Viral RNA kit or a magnetic bead-based method such as the Roche MagNA Pure kit [3]. Reliance on these commercial kits created supply shortages that hindered testing [4]. Traditional laboratory techniques for RNA purification may offer less expensive alternatives to commercial kits. Trizol extraction followed by isopropanol precipitation provides a high yield of purified RNA [5], however, it requires extensive labor, is difficult to scale to high-throughput, and involves hazardous materials. Simpler isopropanol precipitation methods, in which patient swab samples are first mixed with commercial or homemade lysis solutions, have been reported to give Ct values comparable to those obtained using commercial RNA purification kits [6–8].

To obviate the need for RNA purification altogether, several groups have developed protocols for direct addition of swab samples to RT-qPCRs (reviewed in [9]). While this affords a substantial reduction in the time and expense of testing, the absence of a purification step means that RNA is not concentrated, limiting the sensitivity of detection. Moreover, commonly used swab collection solutions may inhibit RT-PCR. Indeed, while several groups have demonstrated RNA amplification by direct addition of swab samples in the widely used viral transport medium (VTM), inhibition of RT-PCR by VTM typically leads to a significant delay in amplification [10–15]. A comparison of commercial master mixes found that the commonly used TaqPath master mix is particularly susceptible to inhibition by VTM [16].

Consequently, researchers have sought other swab collection solutions compatible with direct addition. Commonly used swab collection solutions, including universal transport medium (UTM), M6, or Hank’s medium, have all been shown to work to some extent for direct addition [17–20]. Some RT-PCR-compatible commercial lysis solutions have also been used to detect SARS-CoV-2 by direct addition [21–23], however the high cost of these products may preclude widespread use. An ideal swab collection solution would be widely available or cheaply made in any laboratory, allow for sensitive, direct detection of patient swabs, and not require specialized storage conditions. Proposed swab collection solutions include saline [18], PBS [14], TE [24], or simply distilled water [15, 18]. Also, addition of proteinase K (PK) to UTM or saline was reported to improve detection of viral RNA by direct addition [19, 25].

Finally, most SARS-CoV-2 testing protocols in clinical use or in pre-clinical development rely on commercial one-step RT-qPCR master mixes [5, 13, 18, 19, 26–32]. However, the high cost of commercial master mixes could be prohibitive for widespread testing in resource-limited settings. Master mixes assembled using homemade enzymes may help to address this need [33–35].

The present study evaluates several open-source methods for SARS-CoV-2 diagnostics. A simple isopropanol precipitation protocol provides an effective means of extracting RNA from nasopharyngeal (NP) swab samples that is suitable for subsequent RT-qPCR detection. As an alternative approach, direct addition of small amounts of swab sample in UTM permits SARS-CoV-2 detection, consistent with previous reports, however inhibition of the reaction by UTM limits the amount of sample that can be added, and hence the detection sensitivity. A simple alternative swab collection solution—proteinase K (PK) in water—permits sensitive detection of RNA from *in vitro*-cultured SARS-CoV-2 in contrived swab samples containing human nasal mucus. Finally, a one-step RT-qPCR master mix that can be assembled using homemade Taq polymerase and M-MLV reverse transcriptase (“BEARmix”), permits the detection of as few as tens of RNAs per reaction and can be produced from relatively inexpensive raw materials. Taken together, homemade methods such as these have the potential to circumvent reliance on commercial kits and reagents, lower the cost per test, and facilitate widespread testing.

## Materials and methods

### *In vitro* transcription of N gene RNA

The SARS-CoV-2 N gene sequence was amplified from the N gene control plasmid (Integrated DNA Technologies, Coralville, Iowa, USA, Cat. # 10006625) using primers T7_nCoV_N_F (5' TAATACGACTCACTATAGGGatgtctgataatggaccccaaaatc 3') and M13R (5' caggaaacagctatgaccatg 3'). This was gel-purified using the Zymoclean Gel DNA Recovery Kit (Zymo Research, Irvine, California, USA), and ~70 ng of gel-purified PCR product was *in vitro* transcribed using the HiScribe T7 Quick Kit (New England Biolabs, Ipswich, Massachusetts, USA) in a 20 μL reaction. Following overnight incubation at 37°C, RNA was purified using the RNeasy kit (Qiagen, Hilden, Germany). The concentration of the purified RNA was determined on a NanoDrop spectrophotometer and converted into molar concentration using a calculated molecular weight of 4.2 x 10^5^ g/mol. RNA was diluted to a working stock concentration of 10^6^ molecules per μL, aliquotted, and stored at -80°C.

### Obtaining samples

De-identified nasopharyngeal (NP) swab samples positive and negative for SARS-CoV-2 were obtained from Kaiser Permanente Healthcare, as described in a previous publication [26].

### Inactivation with DNA/RNA shield

For chemical inactivation, NP swab samples and samples of cultured virus were combined with an equal volume of 2x DNA/RNA Shield (Zymo Research, Irvine, California, USA) under BSL3 conditions, mixed thoroughly, and incubated for 20 min at room temperature. Samples were then transferred to new vials prior to being transported out of the BSL3 facility.

### Heat-inactivation and cytopathic effect (CPE) assays

NP swab samples in UTM were heat-inactivated under BSL3 conditions using one of three protocols: 1) 75°C for 30 min, 2) 95°C for 5 min, followed by 75°C for 30 min, 3) 37°C for 30 min in the presence of 0.4 mg/mL proteinase K, followed by 95°C for 5 min and 75°C for 30 min. Samples of cultured virus were inactivated by incubating at 37°C for 30 min (either with or without 0.5 mg/mL proteinase K) followed by 75°C for 30 min. Inactivated samples were subsequently transferred to new vials prior to being transported out of the BSL3 facility.

To confirm complete inactivation, cultured virus was diluted 1:10 into each candidate swab collection solution or water and subjected to heat-inactivation as described above. Inactivated virus was added to cultured Vero E6 cells under BSL3 conditions, and viral infection was assessed by scoring for cytopathic effect (CPE) after 3 days and 7 days. Cells inoculated with non-inactivated SARS-CoV-2 served as a positive control.

### RNA purification using the Qiagen RNeasy kit

RNA was purified from 100 μL of each swab sample using the RNeasy Plus Mini Kit (Qiagen, Hilden, Germany, Cat. #74136). 600 μL of buffer RLT was used in the first step, and RNA was eluted with 50 μL of water in the final step. The gDNA eliminator column step was omitted.

### RNA purification using the QIAmp Viral RNA Mini kit

RNA was purified using the QIAmp Viral RNA Mini kit (Qiagen, Hilden, Germany, Cat. #52906) following the manufacturer's instructions. Briefly, 140 μL of each sample was mixed with 560 μL of buffer AVL containing carrier RNA and incubated for 10 min at room temperature. After addition of 560 μL of 100% ethanol, the samples were passed through purification columns by centrifugation. The columns were washed sequentially with 500 μL of buffer AW1 and 500 μL of buffer AW2, and RNA was eluted using 40 μL of RNAse-free water.

### RNA purification using the MagMax kit

RNA was purified from cultured virus diluted in DNA/RNA Shield using the MagMAX™ Viral RNA Isolation Kit (Thermo Fisher Scientific, Waltham, Massachusetts, USA), following the protocol used for clinical samples in a CLIA-approved lab [26]. 450 μL of each sample was mixed with 10 μL of proteinase K, 10 μL of MS2 phage control, 265 μL of binding buffer, and 10 μL of magnetic beads, and the mixture was incubated at 65°C for 10 min. Beads were pelleted on a magnetic rack and washed once with 750 μL of wash buffer and twice with 500 μL of 80% ethanol, thoroughly resuspending and re-pelleting the beads at each wash step. Ethanol was thoroughly aspirated after the final wash step, and the beads were allowed to air dry at room temperature for 2 minutes. After addition of 25 μL of elution solution to each sample, the beads were thoroughly resuspended by agitation at 1400 rpm in a thermomixer (~3 min) and incubated for a total of 10 min at 65°C. Beads were pelleted again on a magnetic rack, and 20 μL of RNA eluate was withdrawn by pipetting.

### Isopropanol precipitation

Swab samples were inactivated using heat or DNA/RNA Shield as described above. 100 μL of each swab sample was mixed in 1.7 mL microcentrifuge tubes with 0.1 volumes of 3 M sodium acetate, pH 5.2, and 1 μL of 5 mg/mL linear acrylamide. Samples were then mixed with 1.1 volumes of isopropanol, incubated at -20°C for 30 min, and centrifuged at 16,000 *g* for 15 min at 4°C. Supernatants were aspirated, taking care not to disturb the pellets containing RNA. 1 mL of 75% ethanol was added to each sample, and samples were centrifuged again at 16,000 *g* for 5 min at 4°C. Supernatants were carefully but thoroughly aspirated. RNA was redissolved by adding 50 μL of water directly to each pellet and incubating for 10 min at 30°C.

### RT-qPCR with TaqPath master mix

RT-qPCR reactions with TaqPath master mix (Thermo Fisher Scientific, Waltham, Massachusetts, USA) were assembled following the manufacturer's instructions. For a 20 μL reaction, 5 μL of 4x TaqPath master mix was combined with 1.5 μL of SARS-CoV-2 (2019-nCoV) CDC N1, N2, or RNase P qPCR Probe mixture (Integrated DNA Technologies, Coralville, Iowa, USA, Cat. #10006606), RNA sample, and water to a final volume of 20 μL. Volumes were divided by 2 for 10 μL reactions. RT-qPCR was performed on a CFX96 or CFX384 instrument (Bio-Rad Laboratories, Hercules, California, USA) with the following cycle: 1) 25°C for 2 min, 2) 50°C for 15 min, 3) 95°C for 2 min, 4) 95°C for 3 s, 5) 55°C for 30 s (read fluorescence), 6) go to step 4 for 44 additional cycles.

### Enzyme purification and BEARmix reactions

A detailed protocol for purification of Taq DNA polymerase and M-MLV reverse transcriptase and preparation of BEARmix can be found on GitLab: https://gitlab.com/tjian-darzacq-lab/bearmix.

In brief, a 4x buffer + dNTP mixture ("4xBEARbuffer+dNTPs") was prepared containing the following components:

- 200 mM Tris-HCl, pH 8.4
- 300 mM KCl
- 12 mM MgCl_2_
- 40% trehalose
- 40 mM DTT
- 0.4 mM EDTA
- 4 mM each of dATP, dCTP, dGTP, dTTP

Separately, a 100x enzyme mixture ("100x BEAR enzymes") was prepared containing 1.6 mg/mL of homemade Taq DNA polymerase and 0.17 mg/mL of homemade M-MLV reverse transcriptase in storage buffer (50 mM Tris-HCl, pH 8, 100 mM NaCl, 0.1 mM EDTA, 5 mM DTT, 0.1% Triton X-100, 50% glycerol).

For a 10 μL reaction, the following components were mixed on ice:

**Table:** 

4x BEARbuffer+dNTPs	2.5 μL
100x BEAR enzymes	0.1 μL
Primer/probe mixture (containing 6.7 μM of forward and reverse primers and 1.7 μM of TaqMan probe)	0.75 μL
Sample (up to 6.65 μL)	X μL
Water	(6.65—X)μL
Total volume	10 μL

The block of a qPCR machine was allowed to pre-heat to 50°C, and reactions were performed using the following cycle:

1. 50°C for 10 min
2. 95°C for 5 min
3. 95°C for 3 s
4. 55°C for 30 s; plate read
5. Go to 3, 44 additional times

The 95°C incubation in step 2 was extended to 10 min for the experiments shown in Fig 4 and to 10, 15, or 20 min for the hot-start Taq reactions in S7 Fig. A total of 50 rather than 45 cycles were used for the experiments in Fig 4A and S5 and S7 Figs.

### Quantification cycle (Cq) determination by the second-derivative method

Custom MATLAB code (available at https://gitlab.com/tjian-darzacq-lab/second-derivative-cq-analysis) was used to take the numerical second derivative of fluorescence intensity as a function of cycle number, averaged over a 3-cycle sliding window. If the second derivative peak was at the last cycle, then this was taken to be the Cq value. Otherwise, the Cq value was taken to be the center of the second derivative peak, as determined by fitting to a parabola. A user-selected second derivative cutoff was applied to all the samples within each experiment to distinguish amplification from non-amplification.

### Chemidoc imaging of plates

96-well plates and 8-well strips were imaged after PCR using a Chemidoc MP imager (Bio-Rad Laboratories, Hercules, California, USA). The “fluorescein” preset was used to image FAM probe fluorescence with an integration time of 50 ms for Fig 7A and 7B and 100 ms for Fig 7C. For TaqPath reactions, the “rhodamine” preset with was used to image ROX loading control dye fluorescence with an integration time of 500 ms. The fluorescein and rhodamine channel images were overlaid in Fiji (https://imagej.net/Fiji) for Fig 7A and 7B. The tubes in Fig 7C are displayed using the built-in “Green Fire Blue” colormap in Fiji.

### Proteinase K activity assays

Proteinase K (PK) from a frozen aliquot stored at -20°C was diluted to 200 μg/mL in either water or Solution 2 and stored at room temperature or 4°C for 1 to 19 days. As a control, fresh PK was diluted to 200 μg/mL and kept on ice for 15 minutes prior to setting up the reactions. Each reaction contained 4 μL of PK solution, 2 μL of 1 mg/mL BSA in water, and 14 μL of either water or Solution 2. BSA was added last, and reactions were incubated for 1 hour at room temperature. Reactions were stopped after 1 hour by adding 4 μL of SDS loading buffer (200 mM Tris pH 6.8, 400 mM DTT, 10% BME, 8% SDS, 0.4% bromophenol blue, 40% glycerol) and heating the tubes at 95°C for 2 minutes. 12 μL of each reaction was separated on a 4–20% SDS-PAGE gradient gel (Bio-Rad Laboratories, Hercules, California, USA). The gel was fixed and stained with Flamingo Fluorescent protein stain (Bio-Rad) following the manufacturer’s instructions and imaged on a Chemidoc (Bio-Rad) imaging system. Undigested BSA migrates at ~66 kDa, while digestion products migrate below ~30 kDa.

### Preparation and analysis of contrived swab samples

Contrived swab samples were prepared under BSL3 conditions by mixing 3.2 μL of a 3.16 x 10^6^ PFU/mL viral stock (10^4^ PFU) or of a 1:10 dilution of this stock in 1x PBS (10^3^ PFU) to 50 μL of pooled human nasal fluid (Innovative Research, Inc., Novi, Michigan, USA, product # IRHUNF1ML). This mixture was then diluted into 1 mL of either 1x DNA/RNA Shield (Zymo Research, Irvine, California, USA), VCM with 0.4 mg/mL proteinase K (PK), or water with 0.4 mg/mL PK. Control samples were prepared with the same quantities of virus but without nasal fluid. Samples containing PK were incubated at 37°C for 30 min and then heat-inactivated at 75°C for 30 min. RNA was purified using the QIAmp Viral RNA Mini kit, as described above. Alternatively, 1 μL, 5 μL, or 13 μL of each sample was added directly to a BEARmix RT-qPCR. Reactions were performed as described above.

## Results

### Isopropanol precipitation of RNA for SARS-CoV-2 detection

**Fig 1:**
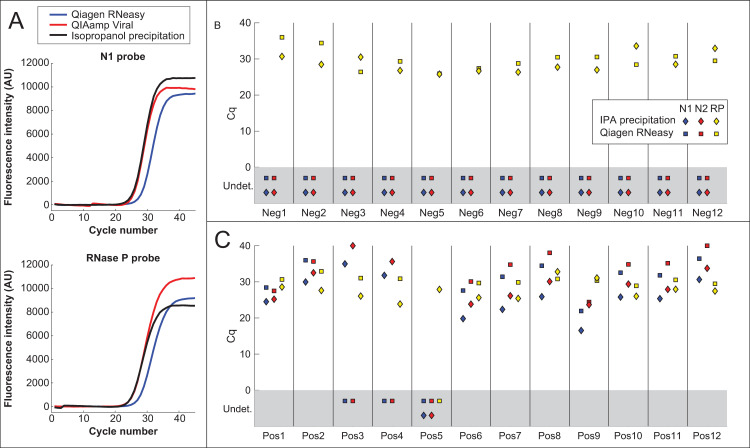
RNA extraction by isopropanol precipitation. **(**A) TaqPath amplification curves of a mixture of human cell RNA and *in vitro*-transcribed SARS-CoV-2 N gene RNA purified using the Qiagen RNeasy Mini kit, QIAamp Viral RNA Mini kit, or isopropanol precipitation. Isopropanol precipitation gives RNA recovery better than the RNeasy kit and comparable to the QIAamp Viral kit. (B-C) Cq values of RNA from positive (Pos) and negative (Neg) NP swab samples in UTM, purified using either the Qiagen RNeasy Mini kit (squares) or isopropanol precipitation (diamonds). Reactions were performed using TaqPath master mix with the CDC SARS-CoV-2 N1 and N2 probes (blue and red points) and the human RNAse P (RP) control probe (yellow points). For points in the gray rectangle, no amplification was observed and Cq values were "undetermined" (Undet).

A simple isopropanol precipitation procedure using inexpensive components (see Materials and methods) was evaluated as an alternative to commercial RNA purification kits. When tested using a mixture of human cell RNA and *in vitro*-transcribed SARS-CoV-2 N gene RNA, isopropanol precipitation gave RNA recovery comparable to the QIAmp Viral kit and significantly better than the Qiagen RNeasy Mini Kit (Fig 1A). Isopropanol precipitation was next evaluated using the same NP swab samples in universal transport medium (UTM) described in a previous publication [26, 36]. For safety, the samples were first inactivated in one of two ways: Samples Pos1-Pos2 and Neg1-Neg2 were mixed with 1 volume of 2x DNA/RNA Shield, while the remaining samples (Neg3-Neg12 and Pos3-Pos12) were treated with 0.4 mg/mL proteinase K for 30 min at 37°C, heated at 95°C for 5 min, and then incubated at 75°C for 30 min. RNA was then purified using either the Qiagen RNeasy Mini kit or isopropanol precipitation (see Materials and methods; note that due to supply shortages, the QIAmp Viral RNA extraction kit was not available when these experiments were performed). Purified RNA was then assayed using CDC-approved Thermo Fisher TaqPath master mix and N1, N2 and RNase P probe sets. Out of 12 known positive samples, 11 showed amplification using isopropanol-precipitated RNA, while only 9 showed amplification using Qiagen RNeasy-purified RNA (Fig 1C; see S1A Fig for comparison to results from ref. [36]). The sample that failed to show amplification by both methods, Pos5, had also shown very high threshold cycle (Ct) values using magnetic-bead based RNA purification (S1A Fig; see also Fig 5A in ref. [36]). The relatively poor performance of the Qiagen RNeasy kit is consistent with a previous report showing that this kit gives higher Cq values than the CDC-recommended QIAamp Viral RNA extraction kit [17]. None of the negative samples showed amplification of viral RNA using either extraction method, while all positive and negative samples (with the exception of sample Pos5 for the RNeasy kit) showed amplification using the human RNase P positive control probe set (Fig 1B and 1C).

### Direct addition of swab samples to RT-PCR reactions

**Fig 2:**
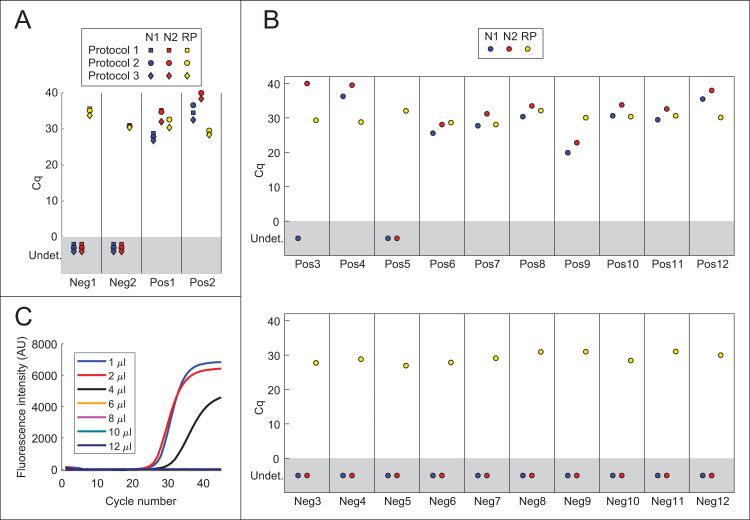
Direct addition of swab samples to RT-PCRs. **(**A) Direct addition of 1 μL of swab sample to 20 μL TaqPath reactions containing N1 (blue), N2 (red), or RNase P (RP; yellow) primer/probe mixtures. Samples were heat-inactivated using one of three protocols: 1) 75°C for 30 min, 2) 95°C for 5 min, followed by 75°C for 30 min, 3) 37°C for 30 min in the presence of 0.4 mg/mL proteinase K, followed by 95°C for 5 min and 75°C for 30 min. (B) Detection of SARS-CoV-2 in positive (Pos) and negative (Neg) NP swab samples by direct addition. Proteinase K was added to each sample to a final concentration of 0.4 mg/mL, and samples were incubated at 37°C for 30 min, 95°C for 5 min and 75°C for 30 min. 1 μL of swab sample was added to 20 μL TaqPath reactions containing N1 (blue), N2 (red), and RNase P (RP; yellow) primer/probe mixtures. (C) Direct addition of different quantities of heat-inactivated swab samples in UTM to TaqPath master mix. The indicated amounts of positive swab sample Pos1 were added to 20 μL TaqPath reactions containing probe N1. See also S1B Fig.

Potentially more useful than simplifying RNA purification would be foregoing RNA purification entirely (see Introduction). A direct RT-qPCR protocol was evaluated in which 1 μL of each swab sample in UTM was added to 20 μL TaqPath reactions containing the N1, N2, and RNase P (RP) probes (Fig 2A). In the interest of safety, aliquots of samples Pos1-Pos2 and Neg1-Neg2 were first heat-inactivated under BSL3 conditions using three different protocols: 1) 75°C for 30 min, 2) 95°C for 5 min, followed by 75°C for 30 min, 3) 30 min at 37°C in the presence of 0.4 mg/mL proteinase K, followed by 95°C for 5 min and 75°C for 30 min. Each protocol includes at least 30 min at 75°C, as this was found to be sufficient to inactivate the virus (S3 Fig). Amplification with primers N1 and N2 was observed for both positive samples (Fig 2A). Neither negative sample showed amplification with primer sets N1 and N2, and RNase P amplification was observed for all samples. Because Protocol 3 gave slightly lower Cq values for viral RNA than Protocols 1 and 2 (Fig 2A), the remaining positive and negative NP swab samples were heat-inactivated using Protocol 3. Each sample was then analyzed by direct addition of 1 μL to 20 μL TaqPath reactions with N1, N2, and RP probes. Amplification was observed using both N1 and N2 in 10 out of 12 positive samples, and 0 out of 12 negative samples (Fig 2A and 2B). Compared to isopropanol-precipitated RNA (cf. Figs 1B, 1C, 2A and 2B), amplification was delayed by on average 4.2 cycles (range 2.3 to 5.8 cycles) for the N1 probe and 3.8 cycles (range -0.9 to 6.8 cycles) for the N2 probe. Sample Pos3, which had the highest Cq values for N1 and N2 using the isopropanol precipitation method, showed very late amplification with N2 but not with N1. All samples showed amplification using the RNase P control probe. Thus, results from direct addition of 1 μL of swab sample were concordant in most cases with results from the standard RNA purification-based assay.

To test whether direct addition of larger quantities of swab sample would yield lower Cq values, 1 μL, 2 μL, 4 μL, 6 μL, 8 μL, 10 μL, or 12 μL of sample were added to a 20 μL TaqPath reaction. Despite the addition of twice as much RNA, Cq values were similar with 2 μL of sample as with 1 μL (Fig 2C and S1 Fig). Furthermore, amplification was inhibited by 4 μL or greater of swab sample. Taken together, these results confirm that viral RNA may be detected by direct addition of swab samples in UTM to TaqPath master mix if the amount of swab sample added does not exceed ~5–10% of the total reaction volume.

### Development of an alternative swab collection solution compatible with RT-PCR

**Fig 3:**
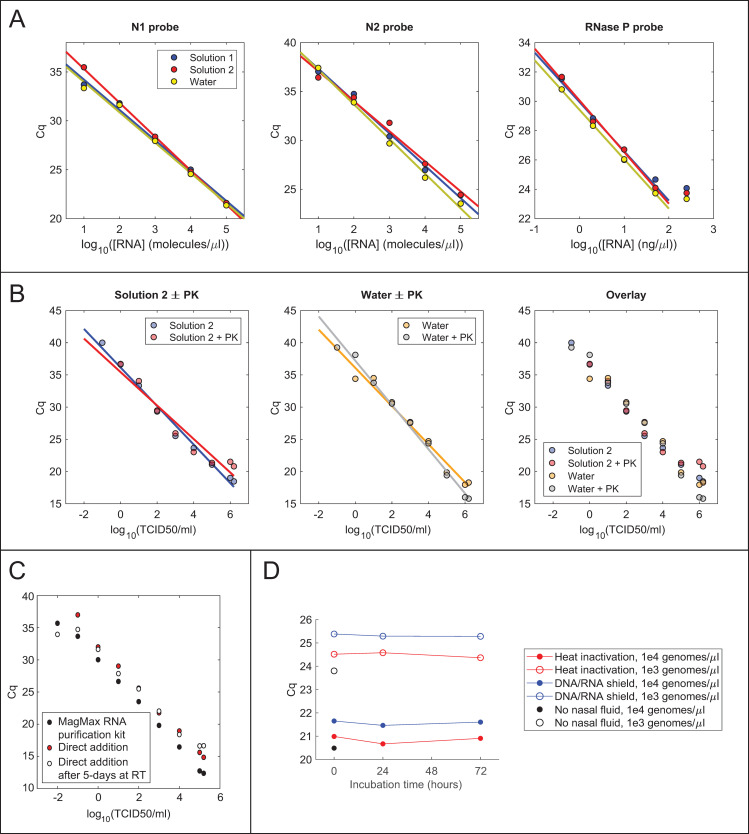
Swab collection solutions optimized for direct addition. (A) RT-qPCR of N gene RNA or human cell RNA in swab collection solutions. RNA was diluted to the indicated concentration in Solution 1 (10 mM Tris, pH 8, 1 mM EDTA, 0.2% Tween 20, 0.2 mM DTT), Solution 2 (10 mM Tris, pH 8, 1 mM EDTA, 0.5% Tween 20, 0.5 mM DTT), or water, and 10 μL of each dilution were analyzed in 20 μL TaqPath reactions containing the indicated probes. (B) Direct addition to RT-qPCR of cultured SARS-CoV-2 heat-inactivated with or without proteinase K treatment in either water or Solution 2 (10 mM Tris, pH 8, 1 mM EDTA, 0.5% Tween 20, 0.5 mM DTT). 13.5 μL of each sample was added to a 20 μL TaqPath reaction. (C) Comparison of viral detection by direct addition or RNA extraction with the MagMax Viral RNA isolation kit (Thermo Fisher). Cultured SARS-CoV-2 was diluted to the indicated number of infectious units into 0.4 mg/mL proteinase K in water. RNA was analyzed using TaqPath master mix and the N1 primer/probe mixture, either by direct addition of 13.5 μL of heat-inactivated sample to a 20 μL reaction or by addition of 5 μL of purified RNA to a 20 μL reaction. (D) Stability of viral RNA in contrived swab samples in PK collection solution. Cq values from TaqPath RT-qPCRs with the N1 probe for virus alone in 1x DNA/RNA Shield (black points) or virus mixed with human nasal fluid, diluted into proteinase K solution, and allowed to incubate for different amounts of time at room temperature prior to heat-inactivation (red points) or inactivation with an equal volume of 2x DNA/RNA Shield (blue points). Results for two different concentrations of virus are shown.

Collecting swab samples in a buffer that does not inhibit RT-qPCR would permit the addition of a greater volume of swab sample per reaction, potentially increasing the sensitivity of the assay. To this end, it was tested whether various additives inhibit TaqPath master mix when 5 μL of each are added to a 10 μL reaction (S2 Fig). Minimal inhibition of TaqPath (ΔCq < 0.5) was seen by 1x TE, 10 mM Tris (pH 7.5, 8, 8.3, or 8.5), ≤ 2 mM EDTA, ≤ 0.5% Triton X-100, ≤ 2% NP-40, ≤ 2% Tween-20, ≤ 2% ICA-630, ≤ 0.02% Sarkosyl, or ≤ 10 mM DTT. Slight inhibition was observed for ≥ 1% Triton X-100 and 0.05% Sarkosyl, while complete inhibition was observed for 1x PBS and ≥ 0.2% Sarkosyl. Based on these results, two candidate solutions were prepared containing non-inhibitory components—Tris-HCl, pH 8, dilute EDTA, Tween-20, and DTT—and 10 μL of *in vitro*-transcribed N gene RNA diluted in either these solutions or water were added to 20 μL TaqPath reactions. Both solutions gave comparable Cq values to water at each RNA concentration, indicating that both are compatible with direct addition to TaqPath master mix (Fig 3A).

To evaluate detection of actual virus by direct addition to an RT-qPCR, serial dilutions of *in vitro*-cultured SARS-CoV-2 were prepared in 1x PBS, and 1 volume of each dilution was mixed with 9 volumes of either water or buffer 2. Because proteinase K treatment gave lower Cq values for NP swab samples, the same mixtures were prepared with 500 μg/mL proteinase K. All samples were incubated at 37°C for 30 min and heat-inactivated at 75°C for 30 min. Complete heat-inactivation of virus in each solution was confirmed using cytopathic effect assays (CPE) in Vero E6 cells (S3 Fig, see Materials and methods). For a given viral dilution, similar Cq values were obtained in all four solutions (Fig 3B). In contrast to NP swab samples, treatment with 500 μg/mL PK did not reduce the Cq values for direct addition of cultured virus (see Discussion).

The above direct addition protocol was compared to the protocol used by the CLIA-approved SARS-CoV-2 testing center at the UC Berkeley Innovative Genomics Institute, which relies on the Thermo Fisher MagMax RNA purification kit [26]. Because RNA is concentrated 18-fold by this purification procedure, it is expected that amplification would be delayed by approximately 2.7 cycles in a direct-addition reaction with 13.5 μL of unconcentrated sample compared to a reaction with 5 μL of purified RNA. Notably, an average delay of 2.5 cycles for the N1 primer was observed (range 2.0 to 3.3), implying that amplification of RNA from crude, heat-inactivated virus is approximately as efficient as amplification of an equivalent amount of purified RNA (Fig 3C).

Because it has been found that proteinase K improves RNA extraction from swab samples, the shelf-life of PK was evaluated in water or Solution 2. PK was diluted to 200 μg/mL in either water or Solution 2 and stored for up to 19 days at room temperature or 4°C. A BSA proteolysis assay was used to measure PK activity either immediately after dilution or after 1, 5, 12, or 19 days of storage (see Materials and methods). PK remained active after 19 days of storage in water at either room temperature or 4°C (S4 Fig). By contrast, PK stored in Solution 2 showed reduced activity after 19 days of storage at room temperature (S4 Fig). These experiments demonstrate that PK may be stored in Solution 2 at 4°C or in water at either 4°C or room temperature for over 2 weeks with no measurable loss of activity.

Finally, the long-term stability of viral RNA was assessed in “contrived swab” samples consisting of human nasal fluid spiked with cultured SARS-CoV-2 and diluted into PK solution. Contrived swab samples were incubated at room temperature for 0, 1, or 3 days and then either heat-inactivated or diluted with an equal volume of 2x DNA/RNA Shield. Analysis of RNA purified using the QIAamp Viral RNA extraction kit showed no increase of Cq value over time, indicating that viral RNA is stable for at least 3 days in PK solution, even in the presence of human nasal fluid (Fig 3D).

### An open-source, one-step RT-qPCR master mix for SARS-CoV-2 detection

In addition to RNA purification kits, commercial RT-qPCR master mixes are an expensive testing component. It would therefore be useful to develop a one-step RT-qPCR master mix consisting of homemade, off-patent enzymes and inexpensive buffer components. After evaluating various enzymes and buffers, the most consistent results were obtained by using a combination of M-MLV reverse transcriptase (specifically, the RNase H-deficient D524N mutant [37]) and Taq polymerase in a buffer containing a high concentration of trehalose. This mixture, dubbed BEARmix (basic economical amplification reaction mix), can be easily prepared just before use by adding an enzyme mixture to a stock solution of buffer and dNTPs.

**Fig 4:**
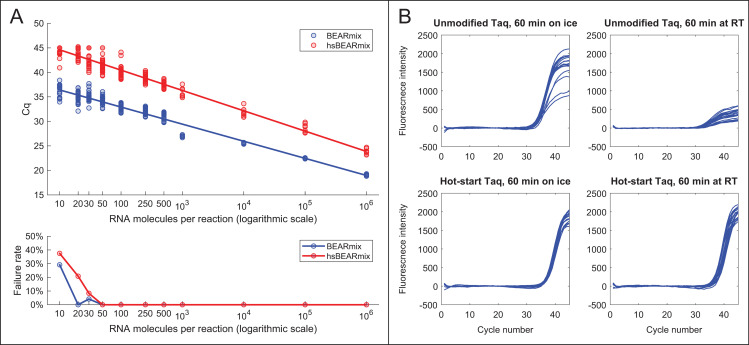
“BEARmix”: An open-source, one-step RT-qPCR master mix. (A) Top panel: Standard curves of BEARmix with unmodified Taq polymerase (blue) and formaldehyde-crosslinked hot-start Taq polymerase (red). Linear fit for non-hot-start BEARmix: Cq = 39.9–3.489x (amplification factor 1.93), where x is the logarithm with base 10 of the number of RNAs per reaction. Linear fit for hot-start BEARmix: Cq = 48.8–4.15x (amplification factor = 1.74). Bottom panel: Fraction of reactions that did not show amplification (failure rate) for each RNA concentration. N = 24 reactions for 10 to 500 copies of RNA, N = 8 reactions for 1000 to 106 copies. (B) Homemade hot-start Taq polymerase permits reaction setup at room temperature. BEARmix reactions were set up using unmodified and hot-start (crosslinked) Taq polymerase with 20 molecules of N gene RNA per reaction. Reactions were performed in a qPCR thermocycler after incubation for 60 min either on ice or at room temperature. In contrast to regular Taq polymerase, amplification by hot-start Taq polymerase is not inhibited by incubating reactions for 60 min at room temperature prior to running the RT-qPCR cycle.

Using dilutions of *in vitro*-transcribed SARS-CoV-2 N gene RNA and the N2 primer/probe set, the expected log-linear relationship was observed between Cq value and amount of input RNA (Fig 4A, upper panel). The limit of detection was approximately 50 RNA molecules per reaction, as amplification was consistently observed for this quantity of RNA but not for smaller quantities (Fig 4A, lower panel and S5 Fig). Raw fluorescence traces without background subtraction exhibited a slow linear increase in baseline fluorescence, even in the absence of template (S6 Fig), which may arise from slow degradation of free probe oligonucleotides by the enzyme mixture. Cq values were thus determined based on the second derivative of fluorescence intensity, which is unaffected by the addition of linear baseline drift ([38]; see Materials and methods and S6 Fig).

A drawback of wild-type Taq polymerase is that it can extend mispaired primers at room temperature, producing “primer dimer” products that compete for amplification with the target amplicon [39–41]. To overcome this problem, companies have generated “hot-start” versions of Taq polymerase, typically by including a proprietary monoclonal antibody or aptamer in the reaction, which inhibits the polymerase at low temperatures but is denatured at high temperature [39–41]. Because these approaches are expensive or patent-protected, an off-patent method was evaluated to convert Taq polymerase to a hot-start version using formaldehyde fixation [42–44]. Treatment with formaldehyde produces crosslinks within the enzyme that inhibit its activity, while incubation at 95°C during the PCR cycle reverses the crosslinks to restore enzymatic activity. Hot-start Taq polymerase prepared in this way was compared with non-crosslinked Taq polymerase in reactions with N gene RNA and the N1 primer/probe set. Reactions were incubated either on ice or at room temperature for various lengths of time after primer addition. Reactions containing unmodified Taq polymerase showed substantially reduced amplification after a 10-minute incubation at room temperature, and amplification was drastically reduced after 1 hour at room temperature (Fig 4B, top row). In contrast, reactions containing hot-start Taq polymerase showed amplification even after a 1-hour incubation at room temperature (Fig 4B, bottom row). However, amplification of a given quantity of RNA with hot-start Taq polymerase occurred at a later cycle than with regular Taq polymerase (Fig 4A and 4B). This was the case across a wide range of input RNA concentrations (Fig 4A), which may reflect incomplete reactivation of the enzyme by heating at 95°C. Consistent with this interpretation, the amplification efficiency, as judged by the slope of the Cq vs. RNA concentration curve, was lower for hot-start Taq than for regular Taq (amplification factor of 1.74 for hot-start Taq versus 1.93 for unmodified Taq). Increasing the time of the 95°C uncrosslinking step to 15 or 20 minutes led to earlier amplification, however amplification with crosslinked Taq was still delayed relative to uncrosslinked Taq (S7 Fig). Thus, formaldehyde crosslinking of Taq permits reaction setup at room temperature, albeit with reduced amplification efficiency.

**Fig 5:**
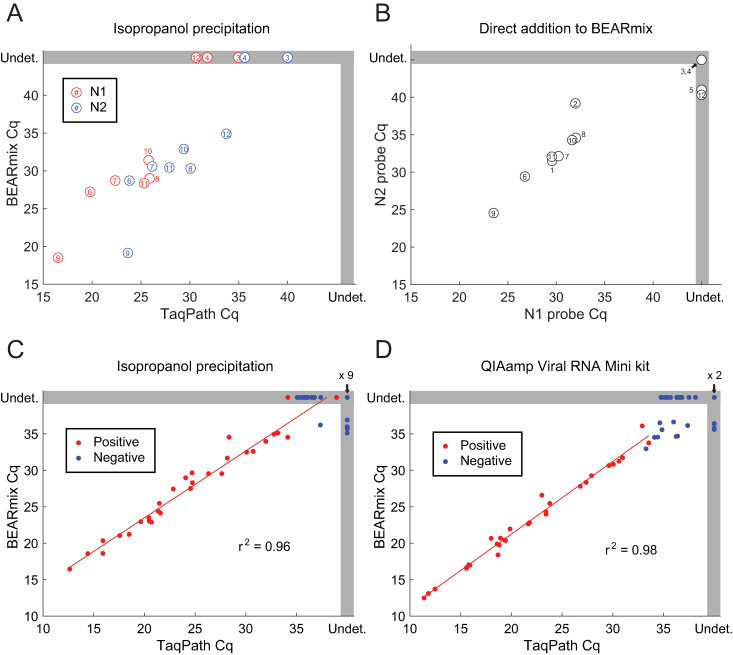
Analysis of clinical samples using BEARmix. (A) Scatterplot of Cq values from RT-qPCR of isopropanol-precipitated NP swab samples using BEARmix and TaqPath. Each circle represents the average of two replicates for an individual positive specimen, with the same numbering as in previous figures. TaqPath Cq values are re-plotted from Fig 1B and 1C. Undet., Undetermined Cq value (no amplification observed). (B) Scatterplot of Cq values for N1 and N2 probes from direct addition of 0.5 μL of clinical swab samples in UTM to 10 μL BEARmix reactions. Each point is the average of a pair of qPCR duplicates. C,D) Cq values of RNA from positive (red points) and negative (blue points) clinical NP swab samples purified using isopropanol precipitation (C) or the QIAamp Viral RNA Mini kit (D) and assayed using the N1 probe with BEARmix or TaqPath.

BEARmix was used to perform RT-qPCR on the remaining isopropanol-precipitated RNA from the NP swab samples that had been analyzed previously using TaqPath master mix (Fig 5A). Amplification with both N1 and N2 probes was observed in 6 out of 9 positive samples tested, and amplification was observed with N2 but not N1 for sample Pos12. Amplification was not observed with either N1 or N2 for samples Pos3 and Pos4, which previously had the highest Cq values with TaqPath master mix. None of the 12 negative samples showed amplification with N1 or N2, while all positive and negative samples showed amplification with the RNase P control probe (mean Cq of 28.3, range 25.4–31.0). Direct addition of 0.5 μL of each swab sample to 10 μL BEARmix reactions gave amplification in at least one of two replicates for 10/12 positive samples and 0/12 negative samples (Fig 5B). However, amplification failed for at least one replicate in three positive samples, while samples Pos3 and Pos4 failed to show amplification in either replicate. Taken together, these results show that RT-qPCR with BEARmix can detect SARS-CoV-2 in clinical samples, either using purified RNA or by direct addition of swab samples, albeit with somewhat lower sensitivity than commercial TaqPath master mix. It is conceivable that sample degradation contributed to the observed reduction in sensitivity in this experiment, as RNA samples were frozen after being assayed with TaqPath, stored at -80°C for 1 week, and thawed for testing with BEARmix.

To further evaluate BEARmix, an additional 30 negative and 30 positive NP swab samples were obtained, which were stored in 1x PBS + 1x DNA/RNA Shield. RNA was extracted using either isopropanol precipitation or the QIAamp Viral RNA Mini kit, and RT-qPCR was performed using the N1 probe and either BEARmix or TaqPath. A strong correlation was observed between Cq values obtained using the two master mixes (Fig 5C and 5D). A slight delay in amplification was observed for BEARmix using RNA purified with the QIAamp kit (1.25 ± 0.14 cycles, mean ± SE) and a greater delay was observed for RNA purified by isopropanol precipitation (3.17 ± 0.23 cycles, mean ± SE). Amplification of IPA-precipitated RNA was delayed relative to QIAamp purified RNA by 2.70 ± 0.29 cycles for TaqPath and 4.50 ± 0.43 cycles for BEARmix. Some of the negative samples showed amplification at late cycles using both TaqPath and BEARmix, suggesting that low levels of cross-contamination may have occurred at some stage of sample processing. Nonetheless, the Cq values of positive and negative samples were largely distinct. Using the lowest Cq value from the corresponding negative samples as a cutoff, amplification was observed in 28 out of 30 positive samples with BEARmix + QIAamp, 27 out of 30 positive samples with BEARmix + IPA precipitation, 29 out of 30 positive samples with TaqPath + QIAamp, and 29 out of 30 samples with TaqPath + IPA precipitation.

### Analysis of contrived swab samples by direct addition to an open-source master mix

**Fig 6:**
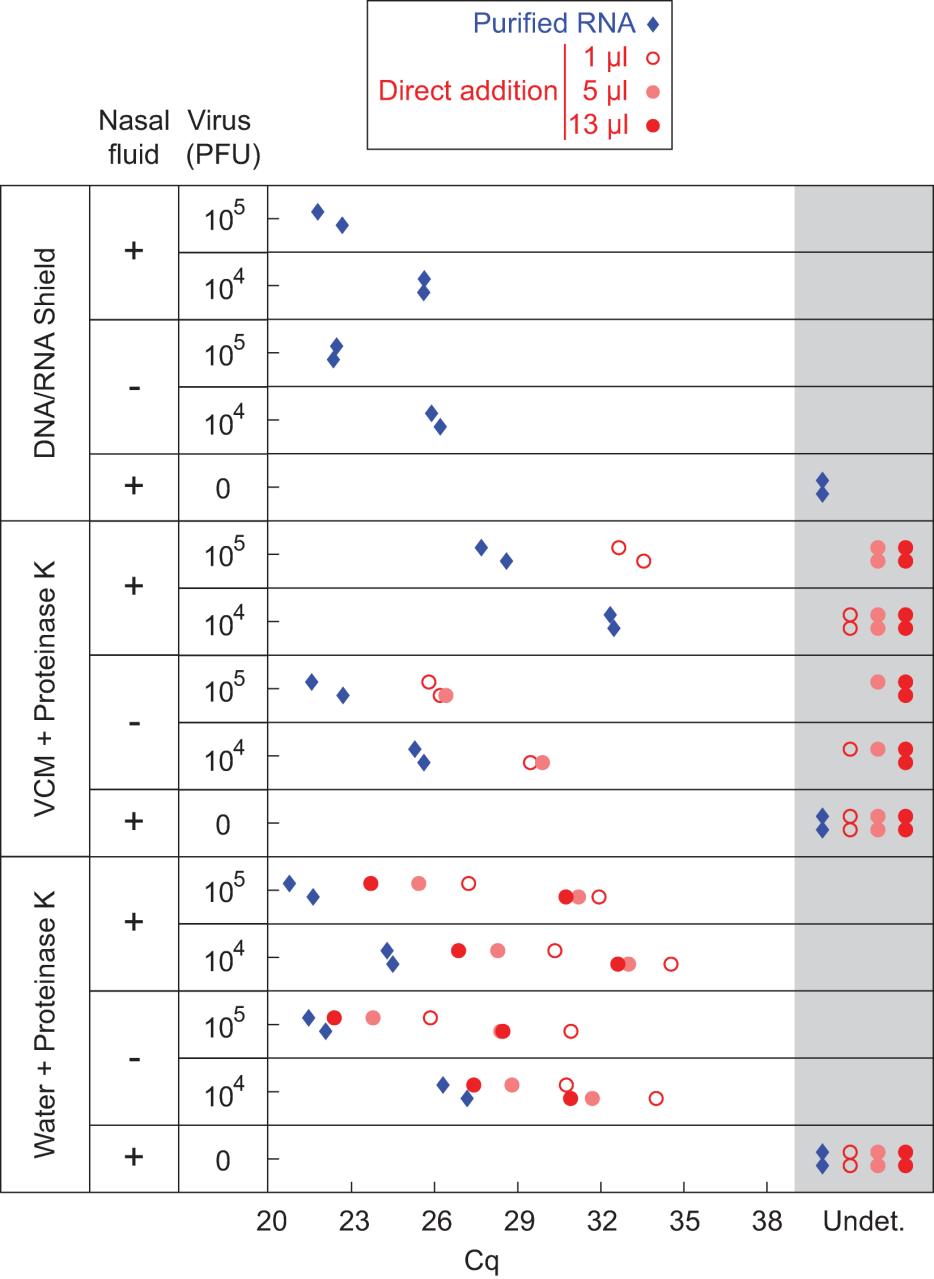
RT-qPCR of contrived swab samples. Indicated quantities of *in vitro*-cultured SARS-CoV-2 were mixed with the swab collection solutions listed in the leftmost column, either alone or in combination with human nasal fluid. Samples were analyzed by RT-qPCR using BEARmix with the N1 primer/probe set either after RNA extraction with the QIAmp Viral RNA purification kit (blue diamonds) or by direct addition (red circles). Two qPCR replicates are shown in separate vertical rows for each condition.

To evaluate a complete protocol in which swab samples are collected into PK solution and then added directly to BEARmix RT-PCRs, contrived swab samples were prepared in which live virus was mixed with pathogen-free human nasal fluid prior to dilution into either DNA/RNA Shield, V-C-M (a Hanks buffered saline-based swab collection solution from Quest Diagnostics similar to VTM) containing 0.4 mg/mL proteinase K, or a solution of 0.4 mg/mL proteinase K in water (Fig 6). Samples in water + PK and VCM + PK were incubated for 30 min at 37°C and then heat-inactivated at 75°C for 30 min.

To assess RNA integrity, viral RNA was extracted from each sample using the QIAmp Viral RNA extraction kit and RT-qPCR was performed using the N1 primer/probe mixture. The presence of nasal fluid did not inhibit RNA amplification for samples in DNA/RNA Shield or water + PK (Fig 6, blue diamonds), indicating that viral RNA is preserved in PK solution in the presence of nasal fluid. However, higher Cq values were observed in the presence of nasal fluid in V-C-M + PK, suggesting that RNA is not preserved as well in this solution in the presence of nasal fluid.

Next, contrived swab samples were analyzed by direct addition to RT-qPCRs. As with samples in UTM (Fig 2C), addition of more than 1 μL of contrived swab sample in V-C-M + PK did not give lower Cq values, but instead inhibited amplification (Fig 6, middle section, red circles). In contrast, addition of increasing amounts of contrived swab sample in water + PK led to lower Cq values (Fig 6, bottom section, red circles). As expected, Cq values were higher for direct addition of contrived swab samples than for purified, concentrated RNA. Thus, while direct addition of swab samples in PK solution provides somewhat lower sensitivity than addition of purified, concentrated RNA, the option to add a larger volume of samples in PK solution improves detection relative to samples in V-C-M, highlighting the key advantage of this method.

### Endpoint detection with a fluorescence imager

**Fig 7:**
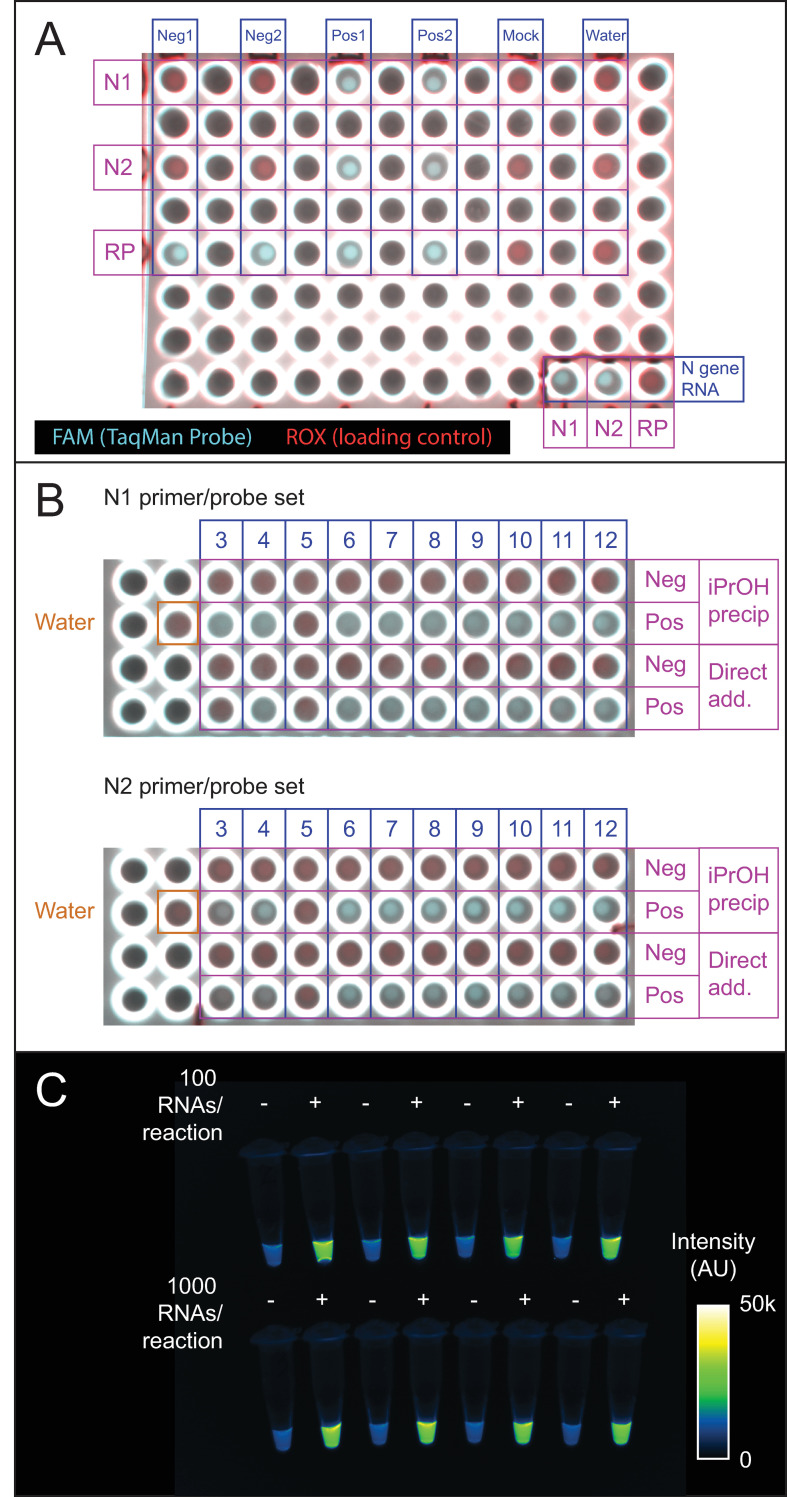
Endpoint fluorescence detection. (A) Endpoint fluorescence image of the qPCR plate used for the first two clinical samples in Fig 1B and 1C. Shown is a 2-channel overlay in which the ROX control dye in TaqPath master mix appears in the rhodamine channel (red) and dequenched FAM product from the TaqMan probe appears in the fluorescein (cyan) channel. An N gene RNA positive control is in the lower right-hand corner. Positive and negative samples are clearly distinguishable based on fluorescence in the FAM channel. Note that leaving empty spaces between samples was an arbitrary choice. B) BioRad Chemidoc fluorescence image of the qPCR plate used for the IPA precipitation and direct addition reactions in Fig 1B, 1C and 2B. Positive and negative samples distinguishable by qPCR are also distinguishable by endpoint fluorescence imaging. Red, rhodamine (0.5 s exposure). Cyan, fluorescein (0.05 s exposure). Scale is set from 0 to 55000 counts for each channel. C) Endpoint detection using the N2 probe set and BEARmix. Reactions were set up in alternating tubes with water (negative control) or *in vitro*-transcribed N gene RNA at 100 or 1000 copies per reaction. An image taken with a 0.1 s exposure time in the fluorescein channel of a ChemiDoc imager (BioRad) is displayed using the Fiji "Green Fire Blue" colormap with lower and upper limits set to 0 and 50000.

Real-time qPCR thermocyclers are expensive instruments, which some testing centers have had to borrow from academic labs [26]. However, standard thermocyclers are relatively inexpensive and ubiquitous, and even less expensive, miniaturized PCR machines have been developed [45]. Visual inspection of images from a standard fluorescence gel imager was evaluated as a means of distinguishing the presence or absence of viral RNA without a real-time fluorescence readout. RT-qPCR plates from the experiments in Figs 2B, 1B and 1C were imaged on a BioRad Chemidoc imager in the fluorescein (TaqMan probe) and rhodamine (loading control) channels (Fig 7A and 7B). Wells that had shown amplification in real-time traces exhibited visibly greater fluorescence in the fluorescein channel from those that had not (Fig 7A and 7B). Similarly, imaging in the fluorescein channel clearly distinguished BEARmix reactions containing 100 or 1000 *in vitro*-transcribed N gene RNAs from negative control reactions without RNA (Fig 7C).

## Discussion

**Fig 8:**
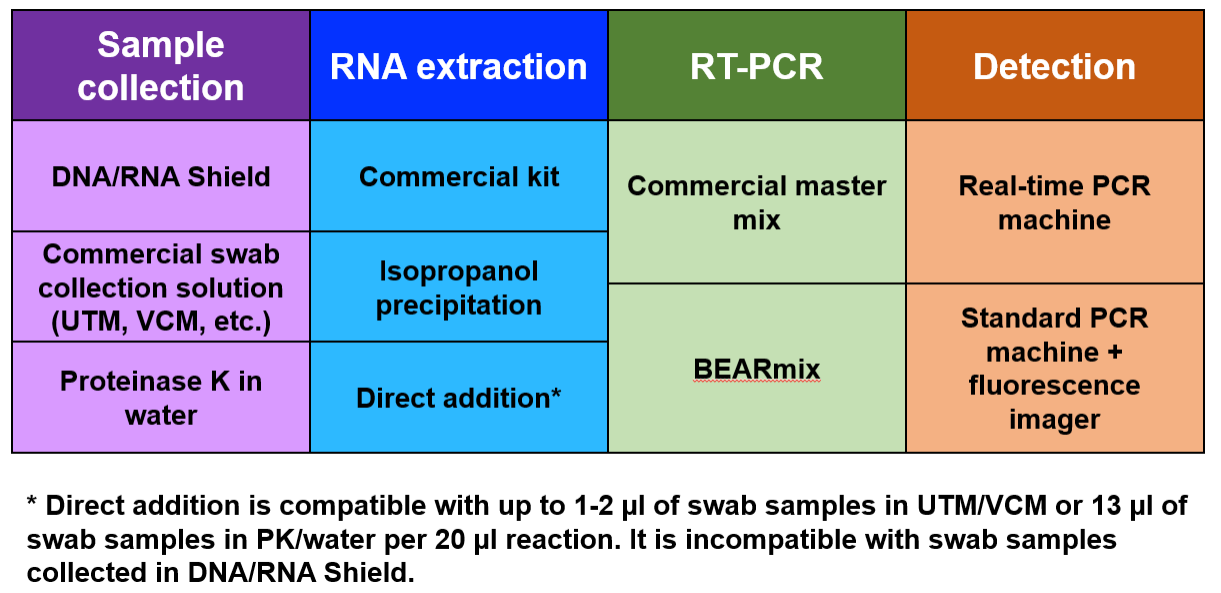
Summary of testing options. Working protocols can be assembled from various combinations of sample collection, RNA extraction, RT-PCR, and detection methods. Economical alternatives are available at each step.

The above results demonstrate that simple, academic laboratory-derived methods for RNA extraction, direct sample addition, and RT-PCR detection provide low-cost alternatives to the use of commercial kits (Fig 8). Adoption of these methods may facilitate widespread testing in a cost-effective way.

Isopropanol precipitation is an extremely simple and inexpensive to extract and concentrate RNA for detection by RT-qPCR (Figs 1 and 5C). While RNA was concentrated between 2-fold and 8-fold in the experiments described above, greater fold concentration could likely be achieved by increasing the amount of input swab sample or decreasing the volume in which the pellet is redissolved. Although recovery yields from isopropanol precipitation were comparable to the QIAamp Viral kit for purified RNA (Fig 1A), isopropanol precipitation gave higher Cq values than the QIAamp kit when tested using NP swab samples in 1x PBS + 1x DNA/RNA Shield (Fig 5C and 5D). Another drawback of this method is that aspiration of supernatants from individual tubes is time consuming and low-throughput compared to plate-based methods (although less time consuming in practice than commercial spin column-based methods). It is possible that precipitating samples in 96-well plates and removing the supernatant using a multi-well aspirator might allow for a greater number of samples to be processed in parallel. Despite these disadvantages, isopropanol precipitation permitted the detection of viral RNA in the majority of positive samples tested (Figs 1C and 5C), establishing it as a possible contingency option if commercial kits are unavailable or unaffordable.

Direct addition of swab samples to RT-qPCR reactions saves money and time by foregoing an RNA purification step. Consistent with previous studies, the above results show that it is possible to detect virus by adding a small volume of heat-inactivated swab sample in UTM to an RT-qPCR (Fig 2). Incubation of swab samples with proteinase K prior to heat-inactivation yielded slightly lower Cq values for detection (Fig 2A). Interestingly, this beneficial effect of PK treatment was not observed for cultured virus (compare Figs 2A and 3B), perhaps reflecting degradation by PK of RNases or some other inhibitory protein component that is present in human fluids but not in cell culture supernatant. Unfortunately, inhibition of RT-qPCR by the commonly used swab collection solutions UTM and V-C-M limits the amount of sample that can be added to the reaction, and hence the sensitivity of detection (Figs 2C and 6). The above results suggest that direct addition would be facilitated by collecting swabs in either a low-salt buffer or water containing proteinase K. Strikingly, direct addition of heat-inactivated virus in low-salt buffer or water gave Cq values close to those expected based on the total RNA copy number, indicating that RT-qPCR amplification is approximately as efficient with heat-inactivated virus as with purified viral RNA (Fig 3C). The direct addition method was likewise effective for contrived swab samples containing cultured virus and human nasal fluid in water + PK, and adding a larger volume of sample generally gave lower Cq values (Fig 6). This method is also safe to use, as the virus can be heat-inactivated without opening the tube (S3 Fig). Samples were heat-inactivated for 30 min in the present study to comply with biosafety regulations, and it is possible that shorter periods of time would be efficacious, however it is critical to inactivate PK completely to avoid inhibition of RT-qPCR. Even with improved collection buffers, there is of course a limit to the volume of sample that can be added per reaction, and direct addition is thus less sensitive than purification methods that concentrate RNA. Testing centers must therefore judge whether reduced sensitivity is worth the time and cost savings of the direct addition method.

Commercial master mixes for one-step RT-qPCR cost up to hundreds of U.S. dollars per milliliter. The results of this study show that “BEARmix”, a simple laboratory-derived master mix, is capable of detecting tens of RNA molecules per reaction. BEARmix is made using M-MLV reverse transcriptase and Taq polymerase, which are easy to purify with high yield in any laboratory equipped for protein biochemistry. A hot-start version of BEARmix can be made by formaldehyde crosslinking Taq polymerase, however this comes with the drawback of less efficient amplification (Fig 4A and 4B and S7 Fig). BEARmix successfully detected SARS-CoV-2 RNA in a majority of NP swab samples, albeit with generally higher Cq values than commercial TaqPath master mix (Fig 5). Validating BEARmix for clinical diagnostics would of course require more extensive side-by-side comparison of BEARmix and a commercial master mix in an actual testing center, and it is likely that the relative performance of BEARmix and other master mixes may differ depending on the primer set used [13]. Additionally, it would be interesting to evaluate BEARmix in combination with direct-addition protocols for saliva testing [46, 47]. This basic master mix recipe could be improved in various ways, for instance, by including dUTP and UDG to prevent amplicon contamination, optimizing the conditions for hot-start Taq preparation and reactivation, or testing other public-domain DNA polymerase and reverse transcriptase variants [48].

Finally, endpoint observations on a fluorescence gel imager are found to provide another means of distinguishing positive and negative samples after RT-PCR. Given that the goal of testing is a binary determination of positive/negative status, rather than absolute quantification of RNA, an endpoint assay of this sort could potentially provide the desired information without an expensive real-time PCR instrument. Alternatively, a hybrid approach could perhaps be used in which reactions are performed on multiple conventional thermocyclers, followed by end-point fluorescence measurements on a real-time thermocycler or fluorescence plate reader.

### Study strengths and limitations

The diagnostic methods described here rely on relatively inexpensive, widely available materials, and it is straightforward to produce the necessary reagents in an academic laboratory. Although the laboratory-derived master mix described here is not quite as sensitive or reliable as commercial master mixes, it successfully detected viral RNA in most clinical specimens tested and showed strong quantitative correlation with a commercial mix. Because the use of a non-hot-start Taq polymerase requires that reactions be prepared on ice, this basic recipe could be improved by developing inexpensive methods to produce more reliable hot-start polymerases. Isopropanol precipitation provides a cheap alternative to commercial RNA purification kits, however it requires tedious manual aspiration of RNA pellets and was found to give higher Cq values for clinical samples than a state-of-the-art commercial kit. Direct addition of swab samples bypasses RNA purification entirely, which greatly simplifies the protocol at the cost of reduced sensitivity. Importantly, collection of swabs into a low-salt solution can boost sensitivity by permitting addition of a larger sample volume per reaction.

## Conclusion

Continued widespread SARS-CoV-2 testing will be crucial to contain the pandemic while vaccines are distributed. This study demonstrates that relatively simple and inexpensive methods can be used to detect SARS-CoV-2 in clinical samples. While these open-source approaches may not match the exquisite sensitivity of expensive commercial kits, testing centers must consider whether some reduction in sensitivity is worth increased availability of tests in the face of economic and logistical constraints. Continued refinement of open-source diagnostic methods and their adoption by “pop-up” testing centers [26, 46, 47] could facilitate expanded testing, both in the current pandemic and in response to novel viruses in the future.

## Supporting information

S1 Fig Analysis of swab samples by isopropanol precipitation and direct addition. A) Comparison of Cq values for isopropanol precipitated swab samples analyzed with TaqPath + probe N1 vs. the mean of the Ct values from three probe sets in a previous publication [1]. B) Direct addition of different amounts of swab sample (related to Fig 2C). The indicated amounts of positive swab samples 1 and 2 ("Pos1" and "Pos2" above), were added to 20 μL TaqPath reactions containing probes N1, N2, and RP.
(TIF)

S2 Fig Effect of additives on TaqPath RT-qPCR. Cq values are shown for 10 μL TaqPath reactions containing 5 x 10^4^ molecules of *in vitro*-transcribed N gene RNA and 5 μL of the specified concentrations of various additives. TX100, Triton X-100. NP-40, Nonidet P-40. T20, Tween 20. ICA-630, Igepal CA-630. Sark, sarkosyl (sodium lauroyl sarcosinate). DTT, dithiothreitol.
(TIF)

S3 Fig Cytopathic effect (CPE) assay for inactivation of SARS-CoV-2. Inactivation experiments were performed in water or Solution 2 (10 mM Tris, pH 8, 1 mM EDTA, 0.5% Tween 20, 0.5 mM DTT), with or without proteinase K. Images were taken 3 days after inoculation of Vero E6 cells with no virus (i), untreated virus (ii), or virus incubated for 30 min at 37°C and 30 min at 75°C in the indicated solutions (iii-vi). Cytopathic effect (CPE) was visible in cultures inoculated with active virus (ii) but not in cultures inoculated with heat-inactivated virus (iii-vi).
(TIF)

S4 Fig Proteinase K activity assay. SDS-PAGE of BSA digestion reactions with proteinase K samples stored under different conditions in water or Solution 2 (10 mM Tris, pH 8, 1 mM EDTA, 0.5% Tween 20, 0.5 mM DTT). Undigested BSA migrates at ~60 kDa (“Undigested”), while proteolysis results in short, digested bands ≤ 30kDa (“Digested”). Lanes from different gels are separated by white space.
(TIF)

S5 Fig BEARmix amplification curves for different input RNA amounts (related to Fig 4A). Curves for individual wells are shown in blue (upper row; non-hot-start BEARmix) or red (lower row; hot-start BEARmix). Means are shown in black. Each column corresponds to a different (average) number of RNA molecules per reaction. Linear background subtraction was performed, using the first 15 cycles to establish the baseline drift.
(TIF)

S6 Fig Second-derivative method for quantification cycle (Cq) determination. Top panel: Fluorescence trace for a BEARmix reaction containing 250 N gene RNA molecules, showing a slow upward drift in baseline fluorescence prior to the onset of detectable amplification. Middle panel: Derivative of fluorescence intensity with respect to cycle number, calculated over a sliding window of ±3 cycles. Bottom panel: Second derivative of the fluorescence intensity, i.e., derivative of the curve in the middle panel. The second derivative is zero during the initial phase of linear baseline drift and peaked near the onset of detectable amplification. Red vertical line: Cq value, determined as the center of a parabolic fit to the peak of the second derivative curve.
(TIF)

S7 Fig Effect of uncrosslinking time on hot-start Taq activity. BEARmix reactions prepared with hot-start Taq (hsTaq) were incubated for 10, 15, or 20 min at 95°C to reverse formaldehyde crosslinks prior to amplification cycles. A 5 min incubation at 95°C was used for regular (non-crosslinked) Taq. Thin curves represent traces for 7 individual reactions, while thick curves represent their average. Longer uncrosslinking times led to earlier amplification, however amplification with hot-start Taq was still delayed relative to unmodified Taq.
(TIF)

S8 Fig Purified enzymes. SDS-PAGE gel of Taq DNA polymerase and M-MLV reverse transcriptase proteins from the final step of purification. Input protein is the eluate from the initial Ni-NTA purification step. FT, flowthrough. MW, molecular weight in kilodaltons.
(TIF)

S1 File (PDF)

## References

[1] Mervosh S, Fernandez M. ‘It’s Like Having No Testing’: Coronavirus Test Results Are Still Delayed. New York Times. 7 Aug 2020. Available: https://www.nytimes.com/2020/08/04/us/virus-testing-delays.html

[2] Larremore DB, Wilder B, Lester E, Shehata S, Burke JM, Hay JA, et al. Test sensitivity is secondary to frequency and turnaround time for COVID-19 surveillance. medRxiv Prepr Serv Heal Sci. 2020. doi: 10.1101/2020.06.22.20136309 33219112 PMC7775777

[3] Centers for Disease Control and Prevention. CDC 2019-novel coronavirus (2019-nCoV) real-time RT-PCR diagnostic panel for emergency use only instructions for use. Atlanta; 2020.

[4] Akst J. RNA Extraction Kits for COVID-19 Tests Are in Short Supply in US. Sci. 2020. Available: https://www.the-scientist.com/news-opinion/rna-extraction-kits-for-covid-19-tests-are-in-short-supply-in-us-67250

[5] Won J, Lee S, Park M, Kim TY, Park MG, Choi BY, et al. Development of a Laboratory-safe and Low-cost Detection Protocol for SARS-CoV-2 of the Coronavirus Disease 2019 (COVID-19). Exp Neurobiol. 2020;29: 107–119. doi: 10.5607/en20009 32156101 PMC7237269

[6] Guruceaga Xabier et al. Fast SARS-CoV-2 detection protocol based on RNA precipitation and RT-qPCR in nasopharyngeal swab samples. medRxiv. 2020. Available: https://www.medrxiv.org/content/10.1101/2020.04.26.20081307v1

[7] Ponce-Rojas Jose Carlos et al. A Fast and Accessible Method for the Isolation of RNA, DNA, and Protein to Facilitate the Detection of SARS-CoV-2. bioRxiv. 2020. Available: https://www.biorxiv.org/content/10.1101/2020.06.29.178384v3.article-metrics 10.1128/JCM.02403-20 PMC8092744 33293367

[8] Wozniak A, Cerda A, Ibarra-Henriquez C, Sebastian V, Armijo G, Lamig L, et al. A simple RNA preparation method for SARS-CoV-2 detection by RT-qPCR. 2020. doi: 10.1038/s41598-020-73616-w 33024174 PMC7538882

[9] Esbin MN, Whitney ON, Chong S, Maurer A, Darzacq X, Tjian R. Overcoming the bottleneck to widespread testing: a rapid review of nucleic acid testing approaches for COVID-19 detection. RNA. 2020;26: 771–783. doi: 10.1261/rna.076232.120 32358057 PMC7297120

[10] Grant PR, Turner MA, Shin GY, Nastouli E, Levett LJ. Extraction-free COVID-19 (SARS-CoV-2) diagnosis by RT-PCR to increase capacity for national testing programmes during a pandemic. bioRxiv. 2020. Available: https://www.biorxiv.org/content/10.1101/2020.04.06.028316v2

[11] Smyrlaki I, Ekman M, Lentini A, de Sousa NR, Papanicoloau N, Vondracek M, et al. Massive and rapid COVID-19 testing is feasible by extraction-free SARS-CoV-2 RT-PCR. medRxiv. 2020. Available: https://www.medrxiv.org/content/10.1101/2020.04.17.20067348v4 32968075 10.1038/s41467-020-18611-5 PMC7511968

[12] Arumugam A, Wong S. The potential use of unprocessed sample for RT-qPCR detection of COVID-19 without an RNA extraction step. bioRxiv. 2020. Available: https://www.biorxiv.org/content/10.1101/2020.04.06.028811v1

[13] Alcoba-Florez J, Gonzalez-Montelongo R, Inigo-Campos A, de Artola DG-M, Gil-Campesino H, Ciuffreda L, et al. Fast SARS-CoV-2 detection by RT-qPCR in preheated nasopharyngeal swab samples. medRxiv. 2020. Available: https://www.medrxiv.org/content/10.1101/2020.04.08.20058495v1 32492531 10.1016/j.ijid.2020.05.099 PMC7833505

[14] Fomsgaard AS, Rosenstierne MW. An alternative workflow for molecular detection of SARS-CoV-2—escape from the NA extraction kit-shortage. medRxiv. 2020. doi: 10.2807/1560-7917.ES.2020.25.14.2000398 32290902 PMC7160440

[15] Hasan MR, Mirza F, Al-Hail H, Sundararaju S, Xaba T, Iqbal M, et al. Detection of SARS-CoV-2 RNA by direct RT-qPCR on nasopharyngeal specimens without extraction of viral RNA. Darlix J-LE, editor. PLoS One. 2020;15: e0236564. doi: 10.1371/journal.pone.0236564 32706827 PMC7380591

[16] Byrnes SA, Gallagher R, Steadman A, Bennett C, Rivera R, Ortega C, et al. Multiplexed and extraction-free amplification for simplified SARS-CoV-2 RT-PCR tests. medRxiv. 2020. 10.1021/acs.analchem.0c03918 33631932

[17] Bruce EA, Huang M-L, Perchetti GA, Tighe S, Laaguiby P, Hoffman JJ, et al. DIRECT RT-qPCR DETECTION OF SARS-CoV-2 RNA FROM PATIENT NASOPHARYNGEAL SWABS WITHOUT AN RNA EXTRACTION STEP. bioRxiv. 2020. doi: 10.1101/2020.03.20.001008 33006983 PMC7556528

[18] Merindol N, Pépin G, Marchand C, Rheault M, Peterson C, Poirier A, et al. SARS-CoV-2 detection by direct rRT-PCR without RNA extraction. J Clin Virol. 2020;128: 104423. doi: 10.1016/j.jcv.2020.104423 32416598 PMC7204723

[19] Marzinotto S, Mio C, Cifu A, Verardo R, Pipan C, Schneider C, et al. A streamlined approach to rapidly detect SARS-CoV-2 infection, avoiding RNA extraction. medRxiv. 2020. doi: 10.1155/2020/8869424 33343767 PMC7727018

[20] Beltrán-Pavez C, Márquez CL, Muñoz G, Valiente-Echeverría F, Gaggero A, Soto-Rifo R, et al. SARS-CoV-2 detection from nasopharyngeal swab samples without RNA extraction. bioRxiv. 2020. Available: https://www.biorxiv.org/content/10.1101/2020.03.28.013508v1.full

[21] Sentmanat M, Kouranova E, Cui X. One-step RNA extraction for RT-qPCR detection of 2019-nCoV. bioRxiv. 2020.

[22] Joung J, Ladha A, Saito M, Segel M, Bruneau R, Huang MW, et al. Point-of-care testing for COVID-19 using SHERLOCK diagnostics. medRxiv. 2020. doi: 10.1101/2020.05.04.20091231 32511521 PMC7273289

[23] Wee SK, Sivalingam SP, Yap EPH. Rapid Direct Nucleic Acid Amplification Test without RNA Extraction for SARS-CoV-2 Using a Portable PCR Thermocycler. Genes (Basel). 2020;11. doi: 10.3390/genes11060664 32570810 PMC7349311

[24] Srivatsan S, Han PD, van Raay K, Wolf CR, McCulloch DJ, Kim AE, et al. Preliminary support for a “dry swab, extraction free” protocol for SARS-CoV-2 testing via RT-qPCR. bioRxiv. 2020. doi: 10.1101/2020.04.22.056283 32511368 PMC7263496

[25] Mallmann L, Schallenberger K, Demolliner M, Eisen AKA, Hermann BS, Heldt FH, et al. Pre-treatment of the clinical sample with Proteinase K allows detection of SARS-CoV-2 in the absence of RNA extraction. bioRxiv. 2020. Available: https://www.biorxiv.org/content/10.1101/2020.05.07.083139v1

[26] IGI Testing Consortium. Blueprint for a pop-up SARS-CoV-2 testing lab. Nat Biotechnol. 2020;38: 791–797. doi: 10.1038/s41587-020-0583-3 32555529

[27] Chandler-Brown D, Bueno AM, Atay O, Tsao DS. A Highly Scalable and Rapidly Deployable RNA Extraction-Free COVID-19 Assay by Quantitative Sanger Sequencing. bioRxiv. 2020.

[28] Kalikiri MKR, Hasan MR, Mirza F, Xaba T, Tang P, Lorenz S. High-throughput extraction of SARS-CoV-2 RNA from nasopharyngeal swabs using solid-phase reverse immobilization beads. medRxiv. 2020.

[29] Zhao Z, Cui H, Song W, Ru X, Zhou W, Yu X. A simple magnetic nanoparticles-based viral RNA extraction method for efficient detection of SARS-CoV-2. bioRxiv. 2020. 10.1016/j.talanta.2023.124479 PMC10035799 36966663

[30] Food and Drug Administration, Coronavirus Disease 2019 (COVID-19) Emergency Use Authorizations for Medical Devices, In vitro Diagnostic EUAs. [cited 9 Feb 2020]. Available: https://www.fda.gov/medical-devices/coronavirus-disease-2019-covid-19-emergency-use-authorizations-medical-devices/vitro-diagnostics-euas#individual-molecular

[31] Xu J, Wang J, Zhong Z, Su X, Yang K, Chen Z, et al. Room-temperature-storable PCR mixes for SARS-CoV-2 detection. Clin Biochem. 2020. doi: 10.1016/j.clinbiochem.2020.06.013 32592724 PMC7313492

[32] Matsumura Y, Shimizu T, Noguchi T, Nakano S, Yamamoto M, Nagao M. Comparison of 12 molecular detection assays for SARS-CoV-2. bioRxiv. 2020. 10.1016/j.jmoldx.2020.11.007 PMC7699162 33259955

[33] Bhadra S, Riedel TE, Lakhotia S, Tran ND, Ellington AD. High-surety isothermal amplification and detection of SARS-CoV-2, including with crude enzymes. bioRxiv. 2020. 10.1128/mSphere.00911-20 PMC8265673 34011690

[34] Bhadra S, Maranhao AC, Ellington AD. A one-enzyme RT-qPCR assay for SARS-CoV-2, and procedures for reagent production. bioRxiv. 2020.

[35] Mascuch SJ, Fakhretaha-Aval S, Bowman JC, Ma MTH, Thomas G, Bommarius B, et al. A blueprint for academic labs to produce SARS-CoV-2 RT-qPCR test kits. J Biol Chem. 2020. doi: 10.1074/jbc.RA120.015434 32883809 PMC7667971

[36] Doudna JA. Blueprint for a Pop-up SARS-CoV-2 Testing Lab. 2020. Available: https://www.medrxiv.org/content/10.1101/2020.04.11.20061424v2 10.1038/s41587-020-0583-3 32555529

[37] Telesnitsky A, Goff SP. RNase H domain mutations affect the interaction between Moloney murine leukemia virus reverse transcriptase and its primer-template. Proc Natl Acad Sci U S A. 1993;90: 1276–80. doi: 10.1073/pnas.90.4.1276 7679498 PMC45855

[38] Rasmussen R. Quantification on the LightCycler. Rapid Cycle Real-Time PCR. Berlin, Heidelberg: Springer Berlin Heidelberg; 2001. pp. 21–34. doi: 10.1007/978-3-642-59524-0_3

[39] Chou Q, Russell M, Birch DE, Raymond J, Bloch W. Prevention of pre-PCR mis-priming and primer dimerization improves low-copy-number amplifications. Nucleic Acids Res. 1992;20: 1717–23. doi: 10.1093/nar/20.7.1717 1579465 PMC312262

[40] Kellogg DE, Rybalkin I, Chen S, Mukhamedova N, Vlasik T, Siebert PD, et al. TaqStart Antibody: “hot start” PCR facilitated by a neutralizing monoclonal antibody directed against Taq DNA polymerase. Biotechniques. 1994;16: 1134–7. Available: http://www.ncbi.nlm.nih.gov/pubmed/8074881 8074881

[41] Dang C, Jayasena SD. Oligonucleotide inhibitors of Taq DNA polymerase facilitate detection of low copy number targets by PCR. J Mol Biol. 1996;264: 268–78. doi: 10.1006/jmbi.1996.0640 8951376

[42] Ivanov I, Löffert D, Kang J, Ribbe J, Steinert K. Method for reversible modification of thermostable enzymes. United States; US6183998B1, 1998.

[43] Hot Start Taq Purification. [cited 9 Feb 2020]. Available: http://tfiib.med.harvard.edu/wiki/index.php/Hot_Start_Taq_Purification

[44] David Long, Medical University of South Carolina, personal communication.

[45] González-González E, Trujillo-de Santiago G, Lara-Mayorga IM, Martínez-Chapa SO, Alvarez MM. Portable and accurate diagnostics for COVID-19: Combined use of the miniPCR thermocycler and a well-plate reader for SARS-CoV-2 virus detection. PLoS One. 2020;15: e0237418. doi: 10.1371/journal.pone.0237418 32790779 PMC7425953

[46] Vogels CBF, Brackney D, Wang J, Kalinich CC, Ott I, Kudo E, et al. SalivaDirect: Simple and sensitive molecular diagnostic test for SARS-CoV-2 surveillance. medRxiv. 2020. Available: https://www.medrxiv.org/content/10.1101/2020.08.03.20167791v1

[47] Ranoa DRE, Holland RL, Alnaji FG, Green KJ, Wang L, Brooke CB, et al. Saliva-Based Molecular Testing for SARS-CoV-2 that Bypasses RNA Extraction. bioRxiv. 2020. doi: 10.1101/2020.06.26.174698 32607508 PMC7325174

[48] Open Bioeconomy Lab: Open Enzyme Collection. [cited 9 May 2020]. Available: https://openbioeconomy.org/projects/open-enzyme-collections/

