# Supplementary figures and images for: Open-source RNA extraction and RT-qPCR methods for SARS-CoV-2 detection
Source: PLoS One. 2021 Feb 3;16(2):e0246647. doi: 10.1371/journal.pone.0246647 (PMC7857565; doi:10.1371/journal.pone.0246647)

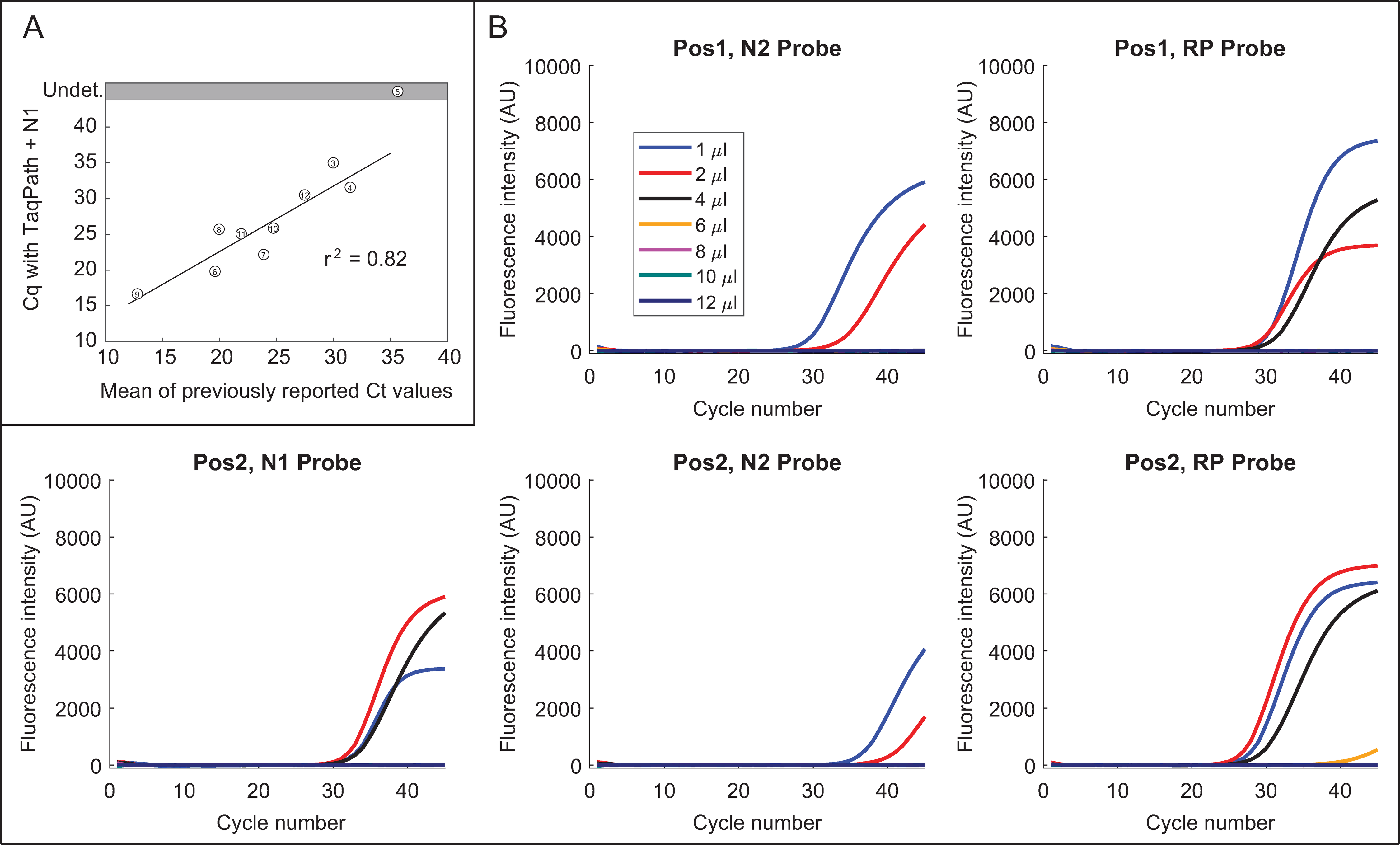

Supplement: S1 Fig — A) Comparison of Cq values for isopropanol precipitated swab samples analyzed with TaqPath + probe N1 vs. the mean of the Ct values from three probe sets in a previous publication [1]. B) Direct addition of different amounts of swab sample (related to Fig 2C). The indicated amounts of positive swab samples 1 and 2 ("Pos1" and "Pos2" above), were added to 20 μL TaqPath reactions containing probes N1, N2, and RP. (TIF) [file pone.0246647.s001.tif]

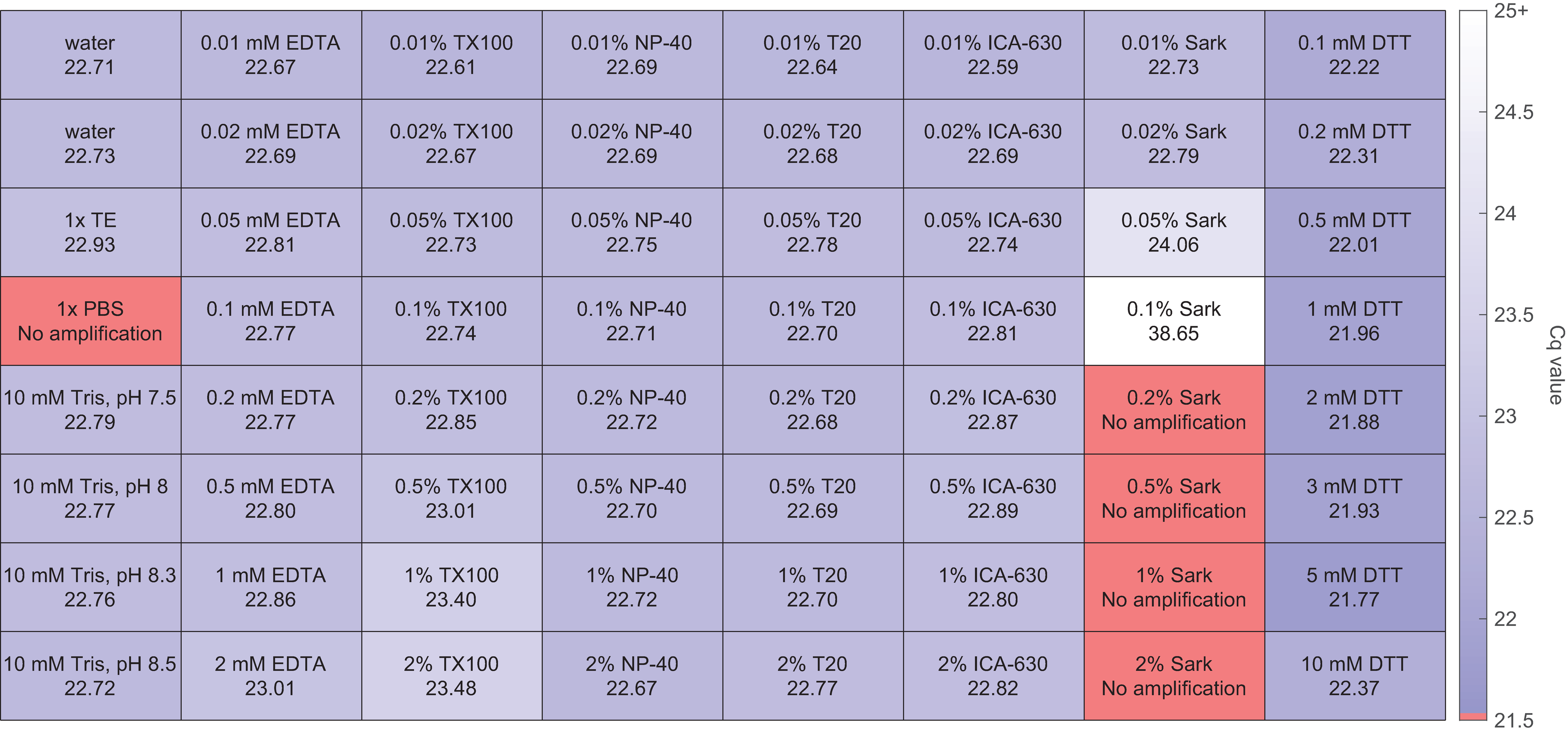

Supplement: S2 Fig — Cq values are shown for 10 μL TaqPath reactions containing 5 x 10^4^ molecules of in vitro-transcribed N gene RNA and 5 μL of the specified concentrations of various additives. TX100, Triton X-100. NP-40, Nonidet P-40. T20, Tween 20. ICA-630, Igepal CA-630. Sark, sarkosyl (sodium lauroyl sarcosinate). DTT, dithiothreitol. (TIF) [file pone.0246647.s002.tif]

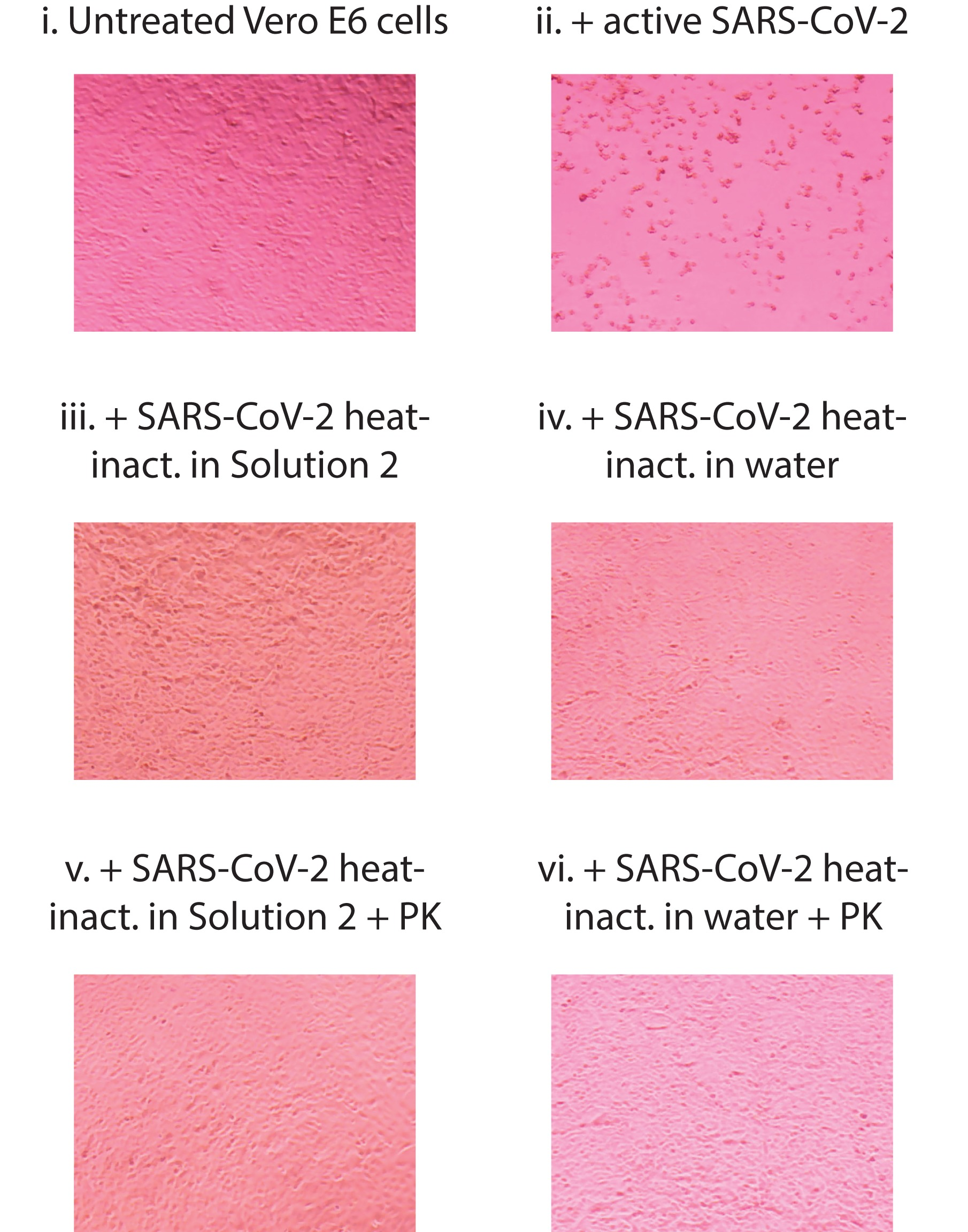

Supplement: S3 Fig — Inactivation experiments were performed in water or Solution 2 (10 mM Tris, pH 8, 1 mM EDTA, 0.5% Tween 20, 0.5 mM DTT), with or without proteinase K. Images were taken 3 days after inoculation of Vero E6 cells with no virus (i), untreated virus (ii), or virus incubated for 30 min at 37°C and 30 min at 75°C in the indicated solutions (iii-vi). Cytopathic effect (CPE) was visible in cultures inoculated with active virus (ii) but not in cultures inoculated with heat-inactivated virus (iii-vi). (TIF) [file pone.0246647.s003.tif]

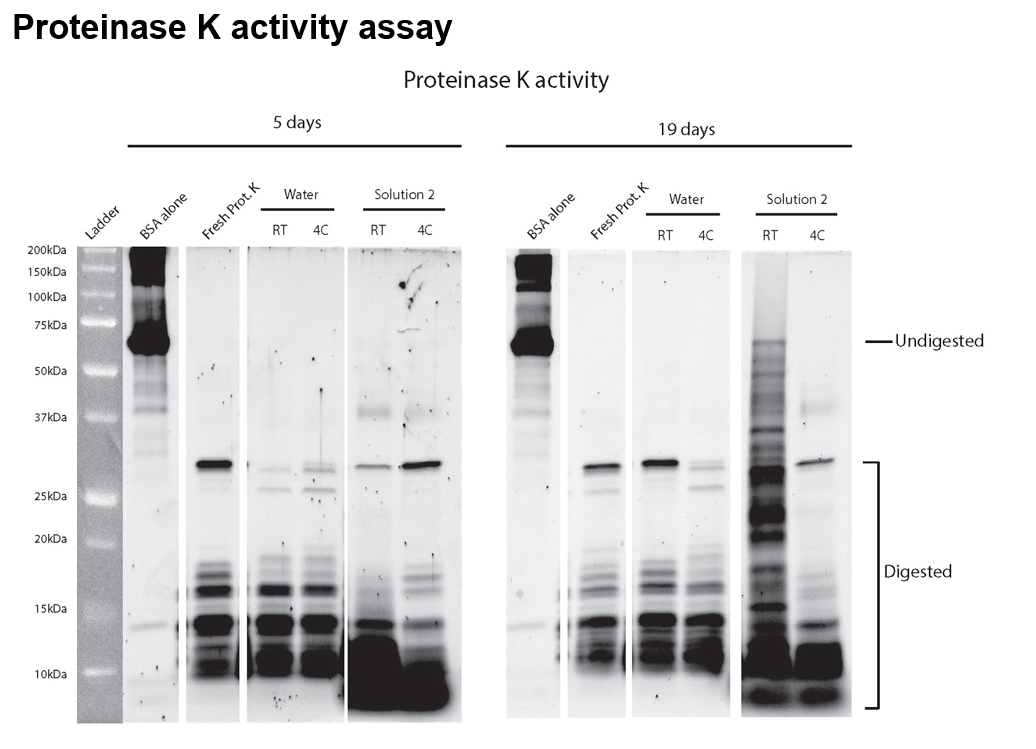

Supplement: S4 Fig — SDS-PAGE of BSA digestion reactions with proteinase K samples stored under different conditions in water or Solution 2 (10 mM Tris, pH 8, 1 mM EDTA, 0.5% Tween 20, 0.5 mM DTT). Undigested BSA migrates at ~60 kDa (“Undigested”), while proteolysis results in short, digested bands ≤ 30kDa (“Digested”). Lanes from different gels are separated by white space. (TIF) [file pone.0246647.s004.tif]

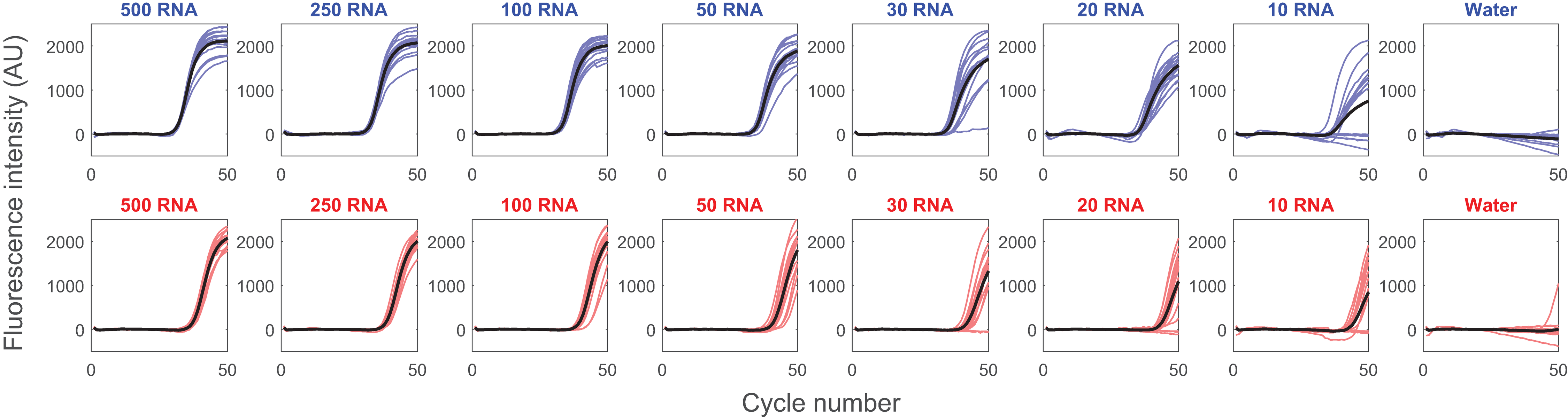

Supplement: S5 Fig — Curves for individual wells are shown in blue (upper row; non-hot-start BEARmix) or red (lower row; hot-start BEARmix). Means are shown in black. Each column corresponds to a different (average) number of RNA molecules per reaction. Linear background subtraction was performed, using the first 15 cycles to establish the baseline drift. (TIF) [file pone.0246647.s005.tif]

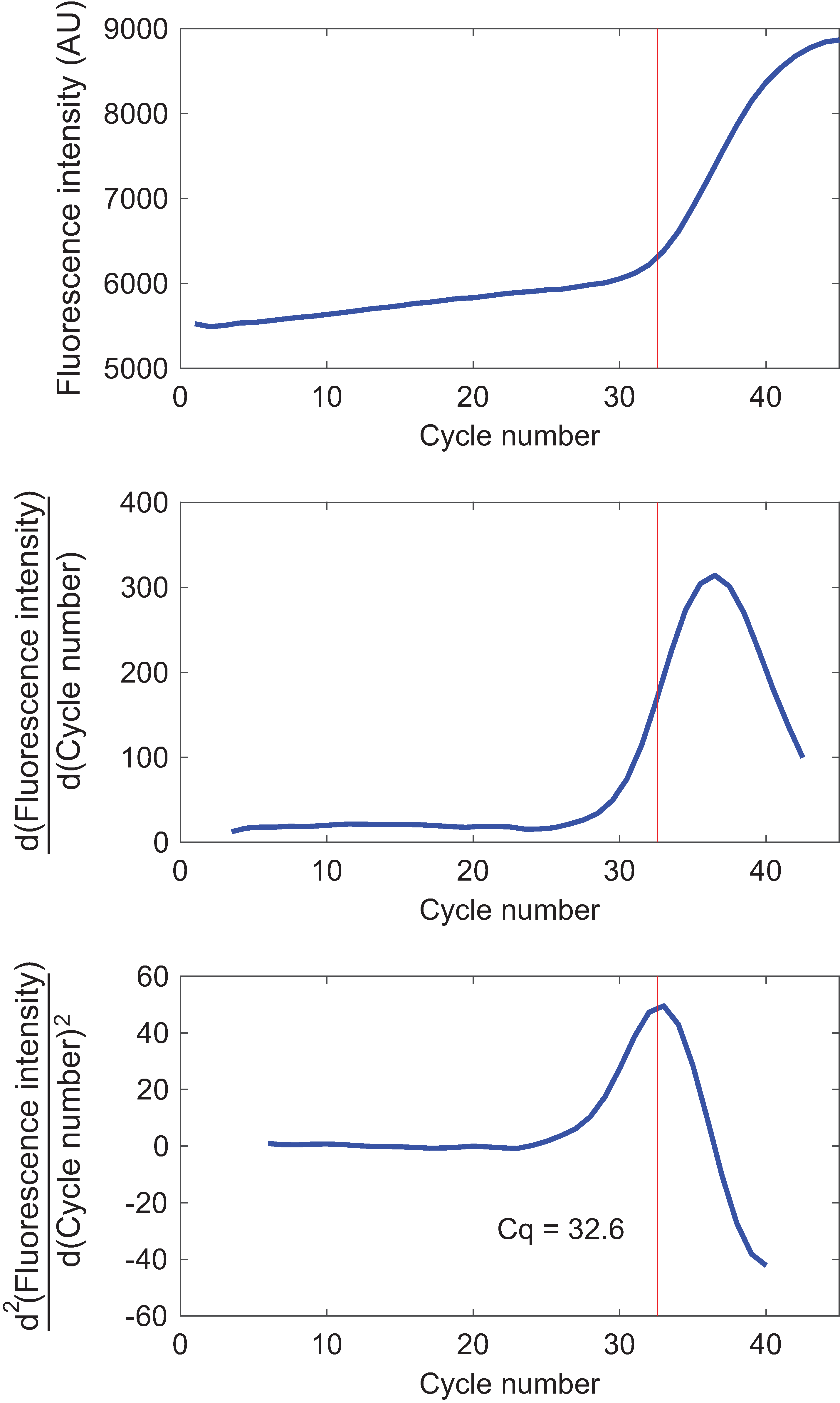

Supplement: S6 Fig — Top panel: Fluorescence trace for a BEARmix reaction containing 250 N gene RNA molecules, showing a slow upward drift in baseline fluorescence prior to the onset of detectable amplification. Middle panel: Derivative of fluorescence intensity with respect to cycle number, calculated over a sliding window of ±3 cycles. Bottom panel: Second derivative of the fluorescence intensity, i.e., derivative of the curve in the middle panel. The second derivative is zero during the initial phase of linear baseline drift and peaked near the onset of detectable amplification. Red vertical line: Cq value, determined as the center of a parabolic fit to the peak of the second derivative curve. (TIF) [file pone.0246647.s006.tif]

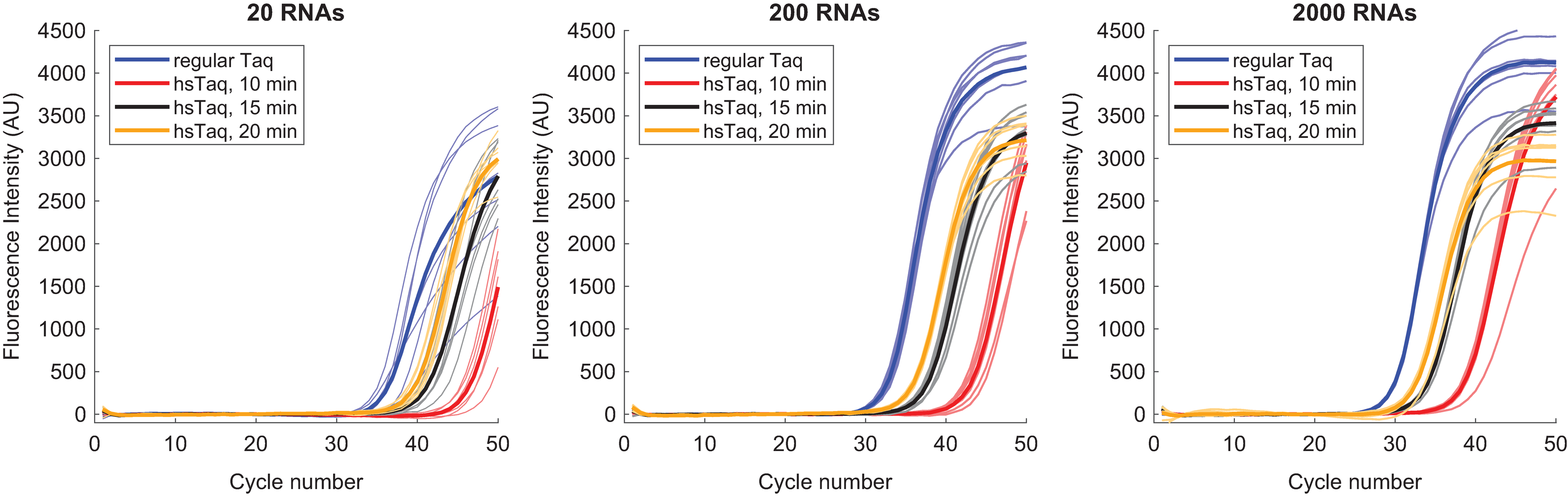

Supplement: S7 Fig — BEARmix reactions prepared with hot-start Taq (hsTaq) were incubated for 10, 15, or 20 min at 95°C to reverse formaldehyde crosslinks prior to amplification cycles. A 5 min incubation at 95°C was used for regular (non-crosslinked) Taq. Thin curves represent traces for 7 individual reactions, while thick curves represent their average. Longer uncrosslinking times led to earlier amplification, however amplification with hot-start Taq was still delayed relative to unmodified Taq. (TIF) [file pone.0246647.s007.tif]

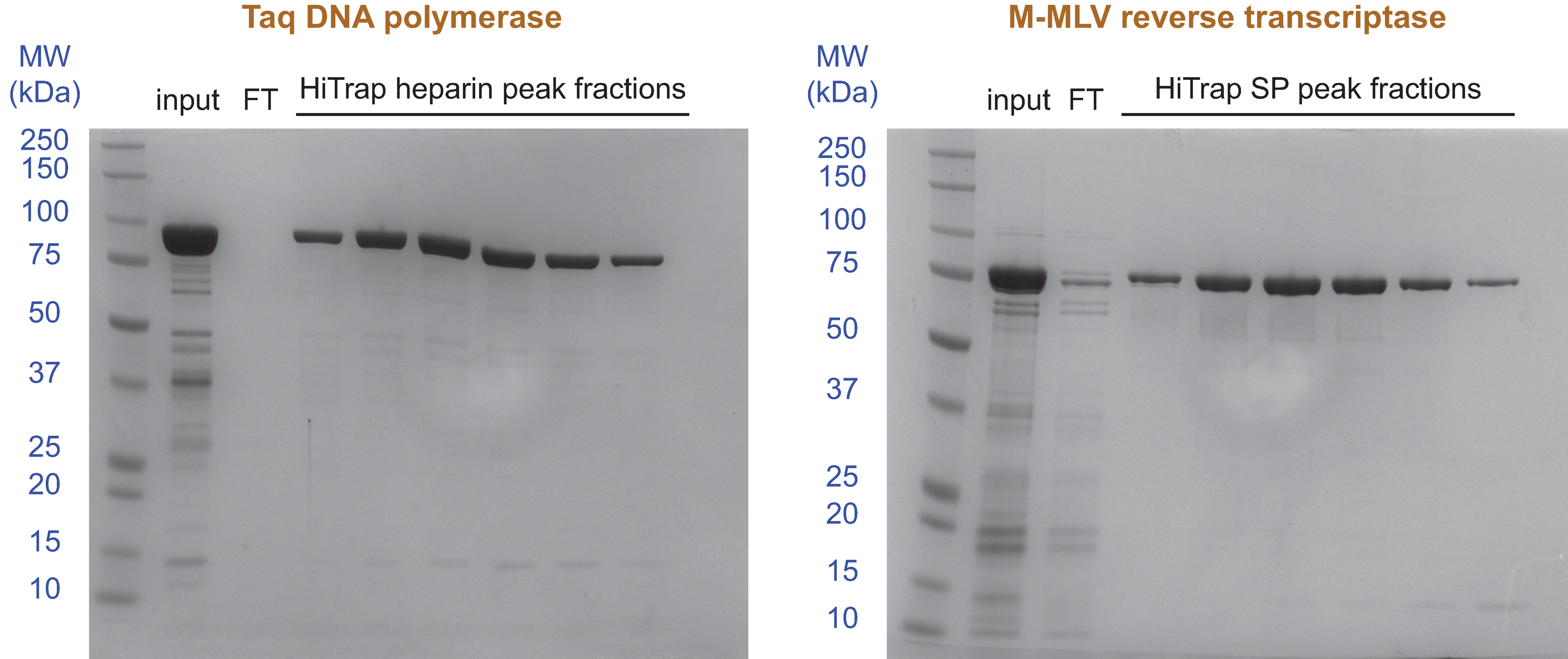

Supplement: S8 Fig — SDS-PAGE gel of Taq DNA polymerase and M-MLV reverse transcriptase proteins from the final step of purification. Input protein is the eluate from the initial Ni-NTA purification step. FT, flowthrough. MW, molecular weight in kilodaltons. (TIF) [file pone.0246647.s008.tif]
