# Supplementary material for: Open-source RNA extraction and RT-qPCR methods for SARS-CoV-2 detection
Source: PLoS One. 2021 Feb 3;16(2):e0246647. doi: 10.1371/journal.pone.0246647 (PMC7857565; doi:10.1371/journal.pone.0246647)
Supplement: S1 File — (PDF) [file pone.0246647.s009.pdf]

Proteinase K Digestion and stability in different buffers after 5 days

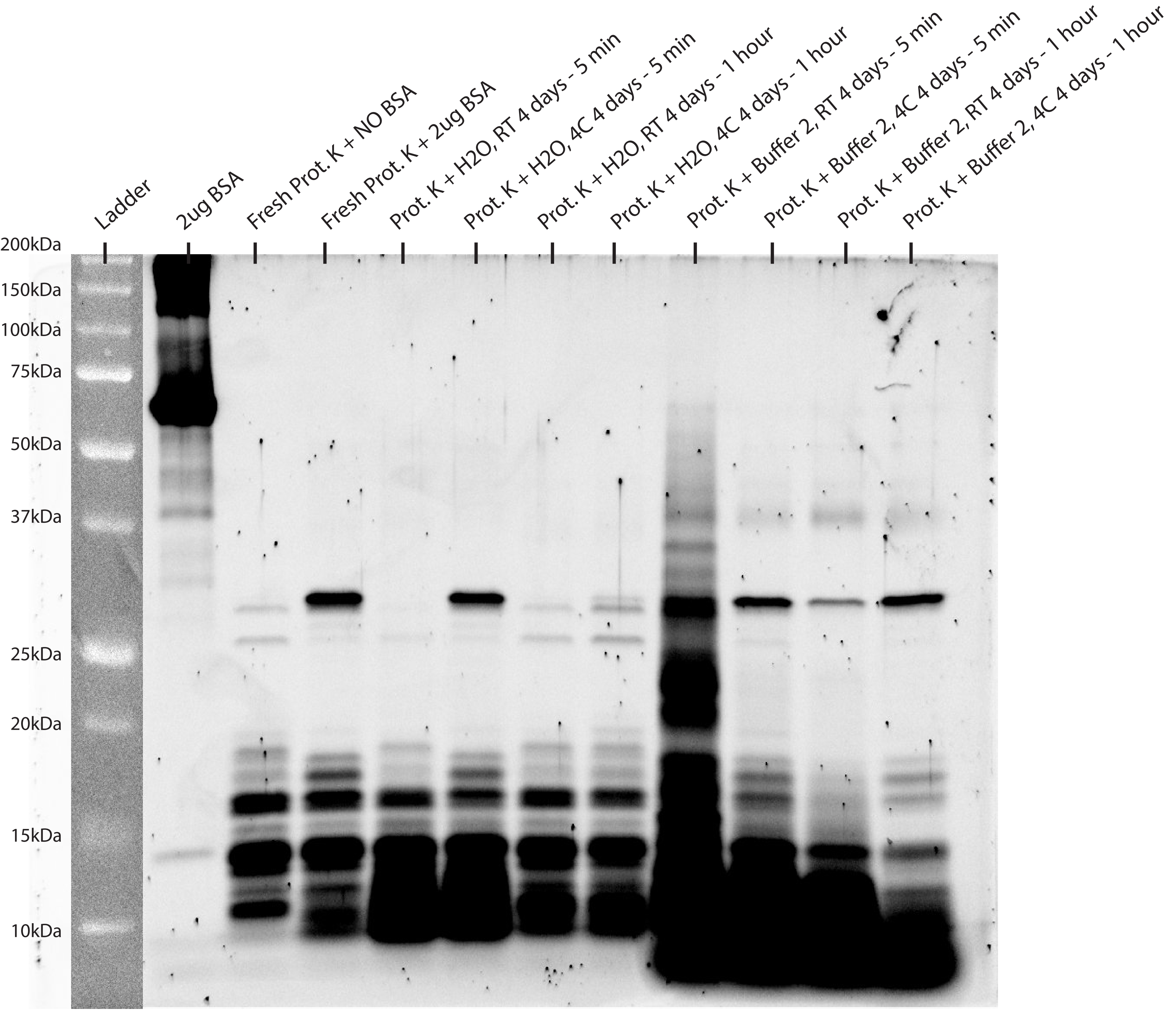

Meagan Esbin, 4/28/2020, Flamingo Stain

Proteinase K Digestion and stability in different buffers after 19 days

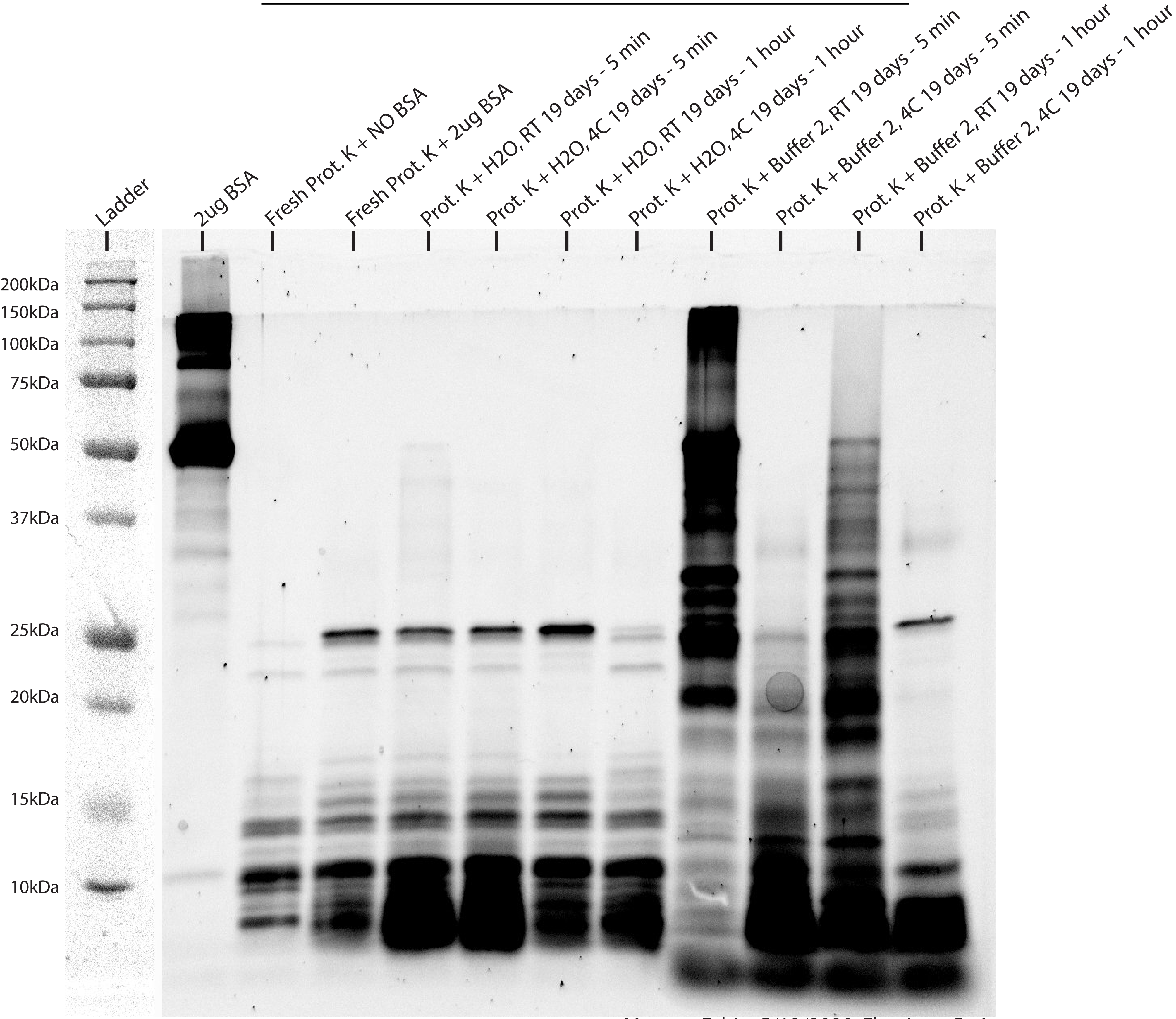

Meagan Esbin, 5/12/2020, Flamingo Stain

Lanes used in Fig. S4:

- Lane 1 – lane 1 in 4/28 gel
- Lane 2 – lane 3 in 4/28 gel
- Lanes 3-4 – lanes 6-7 in 4/28 gel
- Lanes 5-6 - Lanes 10-11 in 4/28 gel
- Lane 7 – lane 1 in 5/12 gel
- Lane 8 – lane 3 in 5/12 gel
- Lanes 9-10 – lanes 6-7 in 5/12 gel
- Lanes 11-12 – lanes 10-11 in 5/12 gel

The other lanes in the 4/28 and 5/12 gels are not shown in the figure.
